# Direct and indirect impact of 10-valent pneumococcal conjugate vaccine introduction on pneumonia hospitalizations and economic burden in all age-groups in Brazil: A time-series analysis

**DOI:** 10.1371/journal.pone.0184204

**Published:** 2017-09-07

**Authors:** Ana Lucia Andrade, Eliane T. Afonso, Ruth Minamisava, Ana Luiza Bierrenbach, Elier B. Cristo, Otaliba L. Morais-Neto, Gabriela M. Policena, Carla M. A. S. Domingues, Cristiana M. Toscano

**Affiliations:** 1 Institute of Tropical Pathology and Public Health, Federal University of Goiás, Goiânia, Goiás, Brazil; 2 Faculty of Medicine, Federal University of Goiás, Goiânia, Goiás, Brazil; 3 Department of Medicine, Pontifical Catholic University of Goiás, Goiânia, Goiás, Brazil; 4 School of Nursing, Federal University of Goiás, Goiânia, Goiás, Brazil; 5 Advisor of the Ministry of Health, Brasilia, Brazil; 6 National Immunization Program, Secretariat for Health Surveillance, Ministry of Health, Brasília, Federal District, Brazil; University of Otago, NEW ZEALAND

## Introduction

Acute lower respiratory tract infections are the leading cause of death in children in low-income regions [1,2]. Pneumonia is the main clinical presentation of such infections, being of high incidence and severity among children and elderly [3,4]. Community-acquired pneumonia is a common cause of hospitalizations among infants throughout the world. It has been estimated that each year, 120 million new episodes of pneumonia, and 11.9 million hospitalizations due to pneumonia occur in children living in low and middle-income regions [5,6].

The economic burden of pneumonia is significant. In recent years, significant economic burden due to pneumonia in children has been demonstrated, in particular in those under two years of age living in low-income countries [7]. *Streptococcus pneumoniae* is responsible for 60% to 75% of all bacterial pneumonia episodes in this age-group [8]. The inclusion of pneumococcal protein polysaccharide conjugate vaccines (PCVs) in National Immunization Programs (NIPs) is recommended as the primary strategy for prevention of pneumococcal disease in low-income countries, because of their substantial effect and cost-effectiveness [9].

Studies assessing the effect of 7-valent PCV (PCV7) introduction on pneumonia epidemiologic and economic burden have demonstrated significant impact shortly after vaccine introduction as a result of averted costs of illness and associated productivity losses [7]. In 2010, PCV7 was replaced by 13-valent (PCV13). The incorporation of these two vaccines in different countries was accompanied by a decrease in the incidence of pneumonia hospitalizations in children soon after vaccine introduction, both in age-groups targeted (direct effect) [10], and non-targeted (indirect effect) by vaccination [11]. Long-term direct and indirect PCV7 impact on pneumonia hospitalizations have also been demonstrated in developed countries [12,13]. The indirect effect of vaccination is attributed to the reduction of nasopharyngeal colonization by vaccine serotypes in vaccinated children, thus contributing to decrease the risk of pneumococcus transmission in the community.

In Brazil, pneumonia hospitalization rates are high throughout all age-groups, with greater burden in children in their first year of life. In March 2010, the Brazilian Ministry of Health introduced 10-valent PCV (PCV10) into its NIP, targeting infants [14]. PCV10 differs from PCV7 and PCV13 not only in numbers of serotypes but also in concentration of the capsular polysaccharides, immunogenicity, carrier proteins, and conjugation process [15]. Shortly after PCV10 introduction, high vaccine coverage rates were observed, reaching 81.7% in 2011, and 94.2% in 2015 [16]. A significant reduction in invasive pneumococcal disease (IPD) was readily observed in 2012 in children under two years of age in most regions of the country [17]. Two recent studies demonstrated the direct impact of PCV10 on the reduction of pneumonia hospitalizations in children in Brazil. The first one demonstrated, by 2011, a significant reduction in pneumonia hospitalizations in children aged 2–23 months in 3 out of 5 large municipalities which are state capitals located in various regions of the country [18]. Another study conducted in 2012, showed nationwide reduction of pneumonia hospitalizations in children younger than one year of age [19]. Despite this evidence, there is still uncertainty regarding the indirect effect of PCV10 on pneumonia hospitalization. In particular, to date, no such study has been conducted in developing countries. Furthermore, the impact of PCV10 on the reduction of the economic burden of pneumonia hospitalizations, which is the main component of such burden, has not yet been demonstrated in developing settings.

In Brazil, most population uses the Unified Health System (SUS), which is provided free of charge. Vaccines are also offered by the NIP through SUS. All hospitalizations occurring nationwide in SUS are recorded in a National Hospitalization Information System (SIH), which includes information on hospital admissions and discharges since 1975 [20,21].

In this investigation, using population-based data from the SIH, we measured both direct and indirect effect on pneumonia hospitalizations in Brazil, after the introduction of PCV10 for infants in 2010. The PCV10 impact on the economic burden of pneumonia hospitalizations in all age-groups was also estimated.

## Methods

### Study design

We conducted an interrupted time-series analysis comparing monthly rates of pneumonia hospitalizations with those of a comparison group of disease. We also conducted an economic burden analysis, modeling the averted costs of pneumonia hospitalizations.

### Study site and population

Brazil is a middle-income country with continental dimensions and with significant social and economic inequalities. Its overall population was estimated at approximately 190 million people in 2010 [22]. The study population considered hospitalization data for all age groups, including children, adults, and elderly, from January 2005 to December 2015. A total of 126,998,568 hospitalizations due to any cause occurred in the SUS during this 11-year period.

### Ethics approval

The Ethics Committee of Federal University of Goiás in Goiania, Brazil, (# 162,532) granted ethical approval for this investigation. Considering we used national bigdata without personal identifiers, the Institutional Research Board (IRB) waived the written individual consent.

### Intervention

PCV10 was introduced in the Brazilian Immunization Program for infants in all municipalities from March to September 2010, being offered as a free of charge universal childhood vaccination [14]. PCV10 includes serotypes 1, 5, and 7F, in addition to serotypes 4, 6B, 9V, 14, 18C, 19F, and 23F, contained in the formulation of PCV7. The adopted vaccination schedule was three primary doses at 2, 4, and 6 months plus a booster at 12 to 15 months of age. During the vaccine introduction period, a catch-up schedule was recommended for children between 7–11 months of age (two doses plus booster), and for those between 12–23 months of age (single catch-up dose). The vaccine was not recommended for children aged 24 months of age and older. Vaccination coverage for the three primary doses reached 81.7%, 88.4%, 93.6%, 92.9% and 94.2% for 2011–2015 respectively [16]. Although PCV7 had been licensed in Brazil in 2001, it was only available to a small portion of the population, through private clinics or through the NIP to high-risk individuals in selected NIP units responsible for providing special vaccines to special target groups.

### Study periods

For the time-series analysis, we defined the pre-intervention period from January 2005 to December 2009, and the post-intervention period from January 2011 to December 2015. The year 2010 was excluded from the analysis, as it was the transition period when coverage of PCV10 increased progressively to over 80%. For the assessment of PCV10 impact on economic burden, we estimated the costs of averted pneumonia cases in the post- intervention period.

### Hospitalization data source

We used data from the SIH of the SUS without personal identification information, which are publicly available online [23]. All hospitalizations funded by SUS, which represents 65.7% of all hospitalizations in the country [24], are recorded into the SIH. International statistical classification and related health problems, 10th revision (ICD10) codes [25] assigned for discharge diagnoses of all hospitalized patients are recorded in SIH. The databases were extracted in April 2017, and the following variables were considered: state of residence, city of residence, date of birth, date of admission, discharge diagnosis, and cost of hospitalization.

### Age-groups and outcomes

For this investigation, the following age-groups were considered: <12 months, 12–23 months, 2–4 years, 5–9 years, 10–17 years, 18–39 years, 40–49 years, 50–64 years, and ≥65 years.

The main outcome of interest was all-cause pneumonia hospitalizations defined by ICD10 codes J12-J18. The comparison groups were a combination of multiple sets of diseases, having as a basis the ICD-10 codes groups proposed by Bruhn *et al*. [26], but performing a manual selection of which ones should be excluded from each age-groups. Excluded disease groups were those potentially influenced by the introduction of PCV-10 vaccine, those whose trend in the pre-vaccination period differed much from the observed trend for pneumonia, those that could have suffered the concurrent effect of another national public health intervention and those that were related to very short-stay or very long-stay hospitalizations. For each age group, a different combination of groups was selected (S1 File).

### Data analysis

#### Descriptive analysis

Rates per 100,000 and numbers of pneumonia and comparison group hospitalizations, and populations were described by year and age-group. Annual average rates of pneumonia and comparison group hospitalizations were examined by period (pre-vaccination and post-vaccination) and age-group.

#### Time-series analysis

Observed hospitalizations rates per 100,000 population were calculated considering in the numerator monthly counts of hospitalization due to specific ICD10 codes listed on the primary diagnosis field, which represents the main or primary discharge diagnosis. Denominators were monthly population estimated by linear regression based of the 2000 and 2010 census data for each of the age-specific populations (the dataset generated is available in S1 Dataset).

The additive Holt-Winters model was used for the interrupted time-series analysis [27–29]. This method provides an exponentially weighted moving average of the observed values in the pre-vaccination. The rationale for using this model is that the most recent observations in the pre-vaccination period will provide the most valuable data to forecast the future. The time series is represented by the model:

$$y_{t}=L_{t}+\;S_{t}+\;T_{t}+\;\xi_{t}$$

where:

*L*_*t*_ represents the Level adjustment component,

*S*_*t*_ represents the Seasonal adjustment component, with *S*_*t*_ = *S*_*t+s*_ = *S*_*t+2s*_ = ⋯, for t = 1, 2, …, (*s*-1) and *s* is the length of the seasonal period.

*T*_*t*_ is the Trend component adjustment,

*ξ*_*t*_ are the residuals.

This model can be forecasted by:

$${\hat{S}}_{t}=\;\gamma \left(y_{t}-{\hat{L}}_{t}\right)+\;\left(\;1-\gamma \right)\left({\hat{S}}_{t-s}\right)\;,\text{with }0\;<\;\gamma \;<\;1$$

$${\hat{L}}_{t}=\;\alpha \left(y_{t}-{\hat{S}}_{t-s}\right)+\left(\;1-\alpha \right)\left({\hat{L}}_{t-1}+{\hat{T}}_{t-1}\right),\text{with }0\;<\;\alpha \;<\;1$$

$${\hat{T}}_{t}=\;\beta \left({\hat{L}}_{t}-{\hat{L}}_{t-1}\right)+\;\left(\;1-\;\beta \right)\left({\hat{T}}_{t-1}\right),\text{with }0\;<\;\beta \;<\;1$$

where α, β and γ are arbitrary constants of initialization: α is the level constant, β is the trend constant and γ is the seasonal constant.

We calculated the initial values of the recurrence equation by:

$${\hat{S}}_{j}=\frac{y_{j}}{\frac{1}{s}\sum_{k=1}^{s}y_{k}}\;,\text{ j}=1,\;2,\;\ldots \;,\text{ s}.$$

$${\hat{y}}_{s}=\frac{1}{s}\sum_{k=1}^{s}y_{k},\text{ and }{\hat{T}}_{s}=0.$$

And the forecast for new values by:

$${\hat{y}}_{t+1}\left(h-1\right)={\hat{L}}_{t+1}+\left(h-1\right){\hat{T}}_{t+1}+{\hat{S}}_{t+1+h-s}\;,\text{ with h}=1,\;\ldots \;,\text{ s}+1.$$

For each of the Holt-Winters models, the trend component was assessed by linear regression and Cox-Stuart tests and the seasonal component by Kruskal-Wallis tests. These indicator variables were only retained in the final models if they were found to be statistically significant.

The error component was obtained by:

$${\hat{\xi }}_{t}=\;y_{t}-{\hat{y}}_{t}$$

i.e. the difference between predicted and observed values. As a measure of prediction accuracy, we present the mean absolute percentage error (MPE) of each model of the pre-vaccination period.[29]

$$MPE=\frac{1}{n}\sum_{t=1}^{n}\left(\frac{y_{t}-{\hat{y}}_{t}}{y_{t}}\right)*100$$

We first fitted Holt-Winters models to the pre-vaccination period, using monthly rates from 2005 to 2009, but excluding the data points from April to October 2009, which corresponded to the period of the pandemic influenza virus A (H1N1) in Brazil. Next, we used Holt-Winters models based on pre-vaccination data to predict the monthly hospitalization rates “expected” to occur in the post-vaccination period (2011–2015), i.e. in the absence of PCV10 vaccination. All models were run separately for the all cause-pneumonia and for the comparison group hospitalizations, for each of the age-groups considered. The post-vaccination predicted rates were then compared to the post-vaccination observed rates, i.e. in the presence of PCV10 vaccination. In order to do that, we calculated the percentage of change between the mean of the observed and the mean of the predicted monthly rates using the following formulae:

$$\text{Percentage Change}=\text{PC}=\frac{\text{MMRobs }-\text{ MMRpred}}{\text{SMRpred}}*100$$

$$\text{Mean of the observed monthly rates}=\text{MMRobs}=\frac{1}{n}\sum_{t=1}^{n}MRobs_{t}$$

$$\text{Mean of the predicted monthly rates}=\text{MMRpred}=\frac{1}{n}\sum_{t=1}^{n}MRpred_{t}$$

We tested the hypothesis that the mean of the observed monthly rates differed from the mean of the predicted monthly rates and calculated a 95% confidence interval for the difference of means, using standard tests for two independent normal populations with unequal and unknown variances.[30]

Finally, we calculated the difference between the percentage changes obtained for the pneumonia and comparison groups, using the formula:

Relative Percentage Change=RPC=PCpneumonia−PCcomparison group

This is our measure of vaccination impact, which we refer to as the “relative percentage change”. Relative percentage changes were calculated considering cumulative years in the post vaccination period (i.e. for 2011, 2011–2012, 2011–2013, 2011–2014 and for the whole post-vaccination period 2011–2015). Respective 95% confidence intervals (95%CI) and p-values were similarly estimated.[30]

For the economic burden, after estimating the predicted annual numbers of pneumonia hospitalization cases in the post-vaccination period, by the age-groups, we calculated the number of all-cause pneumonia hospitalizations averted by vaccination as the difference between the predicted and observed cumulative number of pneumonia hospitalizations in the PCV10 post-vaccination period.

Data management was performed in STATA v. 13.0. We used R software (packages base and stats) for all data analysis and graphics. The modeling scripts are available as supporting information (S2 File).

#### Cost and averted economic burden of hospitalized pneumonia cases

Economic burden analysis considered the SUS perspective. Costs of hospitalized pneumonia cases in all age-groups were obtained by the gross-costing methodology [31], which considers the reimbursement paid by SUS to the hospital in which the individual had been hospitalized. Reimbursement values include both medical and non-medical costs. Medical costs include hospital stay, healthcare professional services and physical therapy, while non-medical costs include hospital stay of a parent or caregiver accompanying the hospitalized individual.

Reimbursed values by cost items are standardized nationwide based on SUS own price list [32]. Hospital stay is valued based on the ICD10 diagnostic code for the main or primary discharge diagnosis. Pneumonia reimbursement value is BRL 504.00 for hospital stay of up to 9 days, after which an additional BRL 20.00 per day is paid. Standard reimbursement values for healthcare professional services for pneumonia are BRL 78.35, which may increase depending on the need for additional specialty professionals. For each physical therapy session, an additional BRL 6.35 is paid. Each day of hospital stay of an accompanying parent or caregiver is reimbursed at BRL 8.00 [32,33].

The average and standard deviations of cost of pneumonia hospitalizations were estimated by age-group, based on reimbursement values by patient reported in the SUS hospitalization database. We assumed that the cost of observed hospitalized cases each year in the period of 2011–2015 was equivalent to the cost of cases averted, should they have occurred.

For the assessment of PCV10 impact on economic burden, we then multiplied the cost per hospitalized pneumonia by the number of all-cause pneumonia hospitalizations averted by PCV10 vaccination, as estimated by the time-series analysis. Costs in BRL were converted to US Dollars (USD) considering the official exchange rate in December 2011 (1 BRL = 0.53 USD), December 2012 (1 BRL = 0.50 USD), December 2013 (1 BRL = 0.43 USD), December 2014 (1 BRL = 0.39), and December 2015 (1BRL = 0.26) [34] Official purchasing power parity exchange rates for 2011 (1 Int$ = 1.47 BRL), 2012 (1 Int$ = 1.56 BRL), 2013 (1 Int$ = 1.65 BRL), 2014 (1 Int$ = 1.73 BRL), and 2015 (1Int$ = 1.87BRL) were used to convert Brazilian Reais into and International dollars (Int$) [35]. We present costs for each year individually and therefore opted not to adjust these amounts to inflation rate [36].

## Results

### Descriptive analysis

From 2005 to 2015, a total of 126,998,568 SUS hospitalizations were recorded. After exclusion of records of hospitalizations due to selected groups of discharge diagnoses (S1 File) and records with missing date of birth (n = 26,716), a total of 78,727,692 records were available for analysis. Among these, pneumonia was recorded as the main discharge diagnosis in 7,829,895 (9.9%) hospitalizations, of which 2,053,419 (26.2%) occurred in children <24 months of age, and 1,902,819 (24.3%) in elderly ≥65 years. Pneumococcal pneumonia was coded as the discharge diagnosis in 0.5% (n = 42,146) of all pneumonia hospitalizations. Considering all age-groups, the total and the average annual number of pneumonia hospitalizations were 3,689,416 and 737,883 in the pre-vaccination period, and 3,378,795 and 675,957 in the post-vaccination periods, respectively (S1 Table).

**Fig 1:**
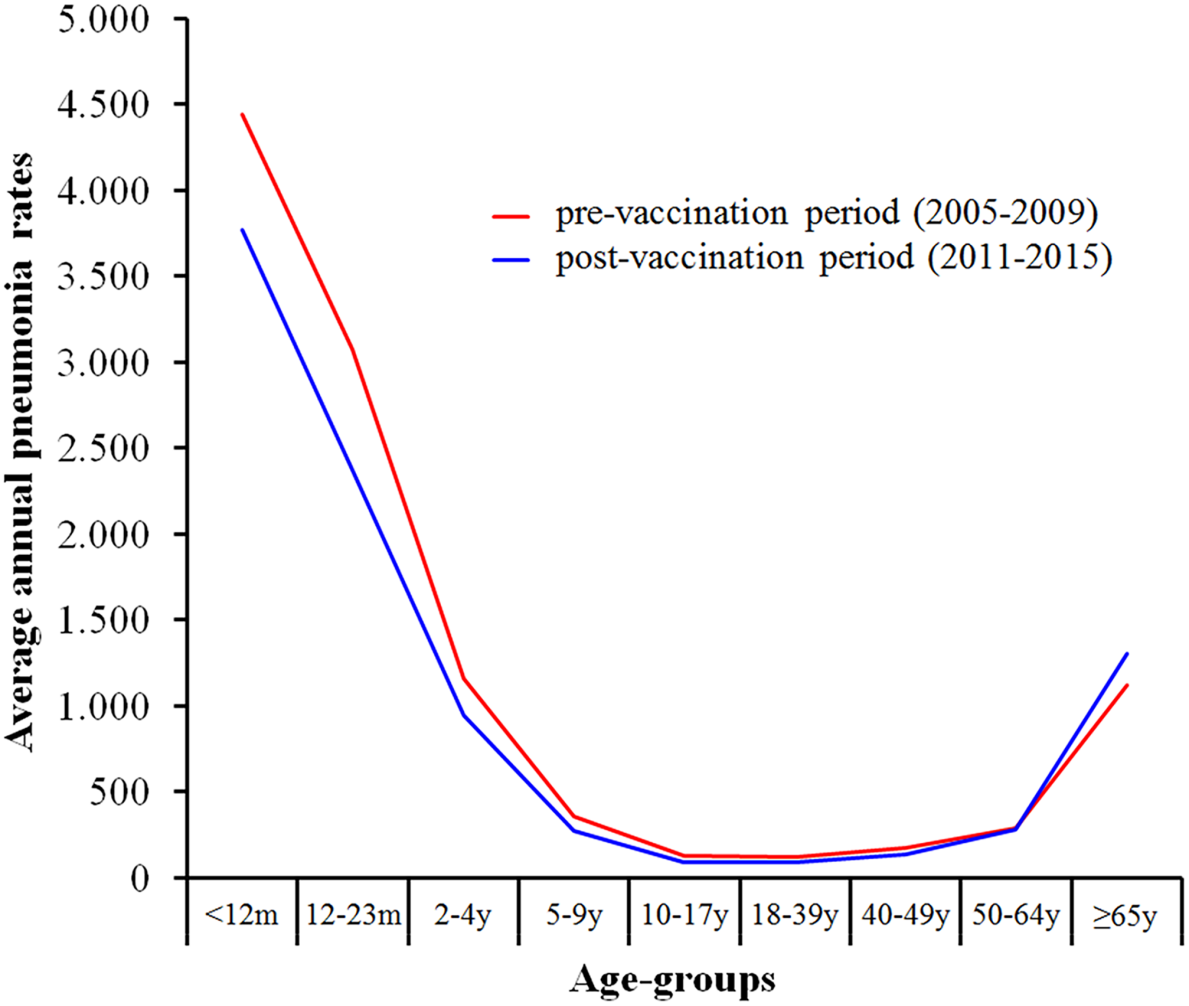
Average annual pneumonia rates (per 100,000 population) of pneumonia hospitalizations in the pre- and post-PCV10 vaccination periods, by age-groups. Brazil, 2005–2015.

Annual rates of pneumonia hospitalization were highest at the extreme age-groups, in both vaccination periods, with rates of approximately 3,500 hospitalizations per 100,000 individuals for both children 2–4 years and elderly (≥65 years). While hospitalization rates declined for children and for adults of less than 50 years of age when comparing the pre- and post-PCV10 periods, there was an increase in hospitalization rates for individuals of 65 years or older (Fig 1). Annual rates of pneumonia and comparison group hospitalizations by age-group are showed in Table 1.

**Table 1:** Annual rates (per 100,000 population) of pneumonia and comparison group hospitalizations, by age-group. Brazil, 2005–2015.

Age-group	2005	2006	2007	2008	2009	2010	2011	2012	2013	2014	2015
**Annual rates for pneumonia hospitalization**											
<12 months	4,335.5	4,489.8	4,378.4	4,260.6	4,741.2	4,170.8	4,067.9	3,864.2	3,879.8	3,589.7	3,433.0
12–23 months	3,043.4	3,151.4	3,098.6	2,933.3	3,159.4	2,959.5	2,568.5	2,435.2	2,389.1	2,284.2	2,175.1
2–4 years	1,120.1	1,168.8	1,182.8	1,106.1	1,219.1	1,161.6	1,066.00	973.7	955.5	904.0	834.5
5–9 years	346.7	370.3	347.4	339.2	389.3	348.5	302.8	281.4	291.3	263.7	231.7
10–17 years	119.5	127.2	119.2	110.0	146.6	122.4	109.5	99.6	100.1	86.5	71.8
18–39 years	122.2	118.7	111.8	105.8	134.9	113.8	101.9	93.3	93.6	82.9	69.3
40–49 years	176.3	174.3	162.3	160.2	189.0	171.9	157.4	147.5	145.3	128.5	114.5
50–64 years	289.2	287.4	277.6	281.1	322.5	315.4	306.1	290.1	294.6	270.7	256.0
≥ 65 years	1,065.8	1,087.0	1,094.9	1,099.7	1,252.2	1,299.2	1,350.6	1,255.8	1,312.2	1,294.2	1,323.5
**Annual rates for comparison group hospitalization**											
<12 months	3,692.70	3,572.24	3,374.46	3,754.10	3,544.69	3,589.19	3,676.66	3,861.75	4,006.76	4,256.58	4,445.58
12–23 months	3,346.47	3,409.78	3,193.84	2,889.16	2,844.65	3,012.17	2,858.62	2,911.11	2,903.42	2,973.37	3,021.94
2–4 years	2,128.40	2,114.45	2,089.03	2,065.32	2,096.00	2,205.25	2,175.76	2,158.80	2,157.00	2,189.48	2,181.04
5–9 years	1,721.63	1,738.47	1,702.33	1,668.77	1,634.86	1,687.35	1,660.92	1,586.38	1,581.47	1,590.94	1,578.99
10–17 years	1,322.61	1,329.06	1,329.18	1,298.41	1,336.50	1,393.71	1,414.56	1,389.27	1,397.54	1,410.59	1,387.92
18–39 years	2,480.91	2,479.97	2,448.47	2,351.05	2,339.60	2,389.49	2,346.15	2,313.43	2,273.34	2,311.41	2,261.11
40–49 years	3,929.34	3,864.94	3,810.36	3,632.64	3,642.10	3,714.82	3,644.20	3,548.95	3,498.91	3,500.17	3,391.60
50–64 years	5,653.54	5,561.73	5,501.90	5,303.96	5,382.94	5,552.73	5,566.27	5,480.72	5,508.29	5,572.26	5,501.74
≥ 65 years	11,231.77	11,077.30	10,819.70	10,287.79	10,543.25	10,815.17	10,779.77	10,615.69	10,732.52	10,867.32	10,926.65

### Time-series analyses

**Fig 2:**
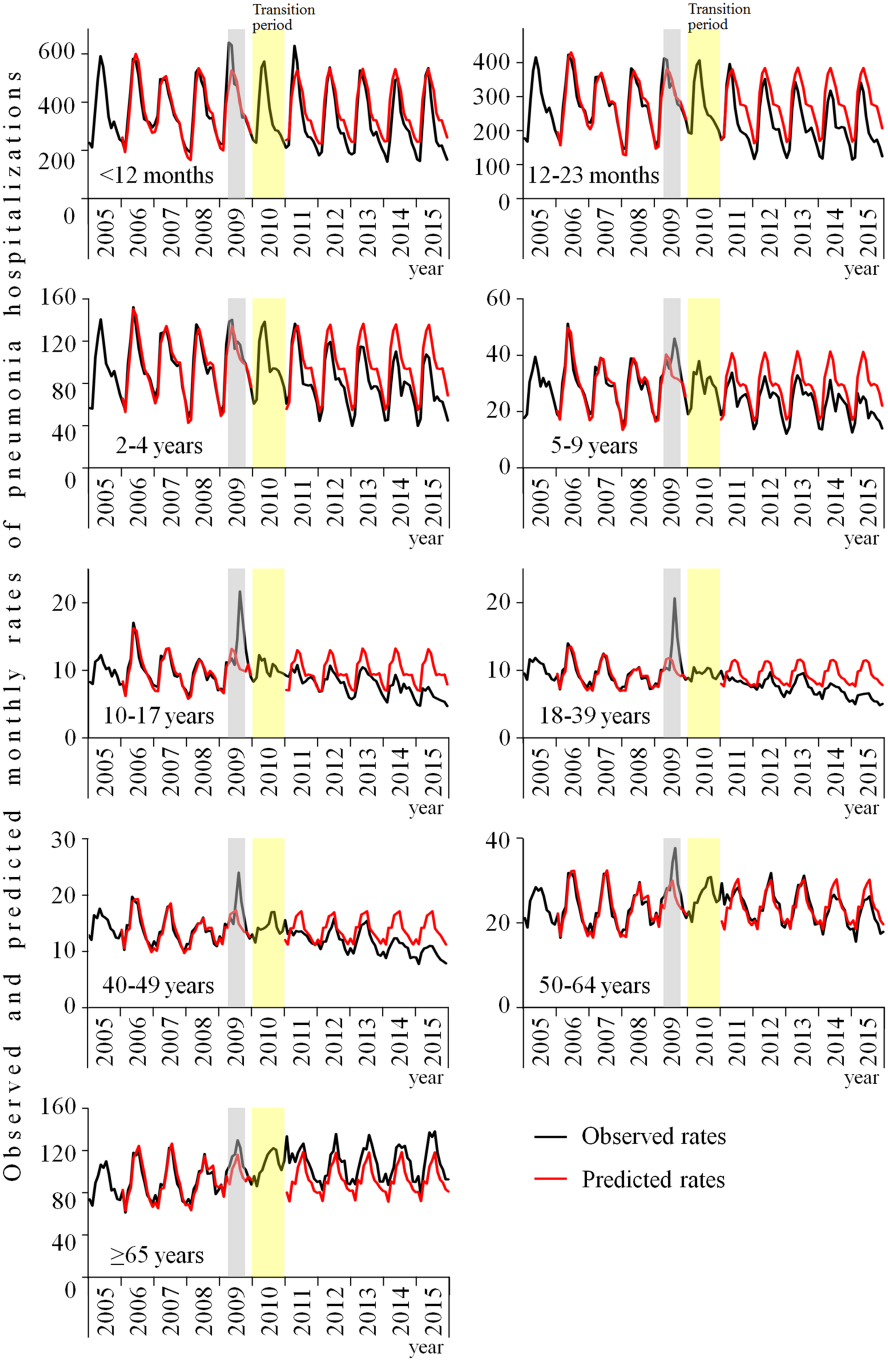
Trends in observed (black lines), fit curve (red lines in the pre-vaccination period), and predicted (red lines in the post-vaccination period) pneumonia hospitalization monthly rates per 100,000 population by age-groups in Brazil. Pre-vaccination period: 2005–2009; Transition period: 2010 (year of PCV10 introduction); Post-vaccination period: 2011–2015. Routine infant PCV10 vaccination was introduced through March to September 2010 by the National Immunization Program. The yellow bar represents the transition period which was excluded from the time-series analysis. Gray bar highlights the months excluded of the flu pandemic months (April-October 2009) from the model.

For the purpose of the time-series analysis, after excluding hospitalizations occurring during the H1N1 influenza pandemic in Brazil (n = 4,229,226), a total of 74,498,466 hospitalizations were considered. Trends of observed (black lines) and predicted (red lines) rates of pneumonia hospitalization, by age-group, for the entire study period are presented in Fig 2. For almost all age-groups, but particularly for those aged 10 to 64 years, it is possible to visualize a peak of hospital admissions in 2009, overlapping with the influenza pandemic months in Brazil (gray vertical bands). Fig 2 shows that in the post-vaccination period, observed rates of pneumonia hospitalizations were lower than predicted rates for all age-groups which are shown with their 95% confidence intervals, except for elderly (≥ 65 years). The differences between observed and predicted rates increases significantly over time in all age groups, except for those aged 65 years and older. The mean percentage error of each model of the pre-vaccination period, which is a measure of model fitness, is presented in S2 Table.

Trends of observed and predicted rates of comparison group hospitalizations are displayed in S1 Fig. Of note, for the youngest age-group, rates are increasing in the post-vaccination period.

Considering the whole post-vaccination period, a significant decrease in the estimated percent of change in pneumonia hospitalization rates following PCV10 introduction is demonstrated in all age groups up to 49 years of age, varying from 13.9% to 17.6% in age-groups targeted by vaccination, and 16.8% to 20.9% in the 10–49 year age-group, not targeted by vaccination (Table 2). A non-significant reduction was found for individuals aged 50–64 years. PCV10 vaccination was not shown to decrease pneumonia hospitalization rates in individuals aged 65 years and more; on the contrary, a significant increase (16.6%, p<0.001) in rates was observed in the post-vaccination period compared to the pneumonia predicted rates. After taking into consideration the percentage of change in hospitalizations of the comparison group, the impact of vaccination, expressed as a relative percentage difference, for the target population ranged from 17.4% to 26.5%. In individuals 10–49 years of age, not target for immunization, a significant decrease in pneumonia hospitalizations was observed with impact ranging from 11.1% to 27.1% (Table 2).

**Table 2:** Percentage change in rates for pneumonia hospitalization and comparison groups, and relative percentage change (PCV10 impact) with respective confidence intervals, by age-group. Brazil, 2011–2015.

Age-group	% of change	95% CI	P-value
**Pneumonia**			
<12 months	-13.9	-22.9; -4.9	0.017
12–23 months	-22.2	-30.7; -13.8	0.000
2–4 years	-17.6	-26.0; -9.3	0.000
5–9 years	-21.8	-30.2; -13.5	0.000
10–17 years	-20.9	-27.9; -14.0	0.000
18–39 years	-21.4	-27.0; -15.9	0.000
40–49 years	-16.8	-21.9; -11.7	0.000
50–64 years	-1.1	-5.5; 3.3	0.696
≥65 years	16.6	12.4; 20.8	0.000
**Comparison group**			
<12 months	12.6	10.9; 14.2	0.000
12–23 months	-4.9	-7.4; -2.4	0.000
2–4 years	3.9	2.3; 5.4	0.000
5–9 years	-5.1	-6.8; -3.4	0.000
10–17 years	6.2	4.7; 7.7	0.000
18–39 years	-4.2	-5.8; -2.6	0.000
40–49 years	-5.7	-7.6; -3.8	0.000
50–64 years	1.8	0.2; 3.5	0.043
≥65 years	1.4	-0.3; 3.0	0.143
**Relative percentage change**			
<12 months	-26.5	-35.5; -17.5	0.001
12–23 months	-17.4	-25.8; -8.9	0.006
2–4 years	-21.5	-29.8; -13.2	0.002
5–9 years	-16.8	-25.1; -8.4	0.006
10–17 years	-27.1	-34.1; -20.2	0.009
18–39 years	-17.3	-22.8; -11.7	0.006
40–49 years	-11.1	-16.2; -6.0	0.004
50–64 years	-3.0	-7.4; 1.5	0.730
≥65 years	15.2	11.0; 19.4	0.005

All analysis excluded data from influenza pandemic months (April-October 2009).

**Fig 3:**
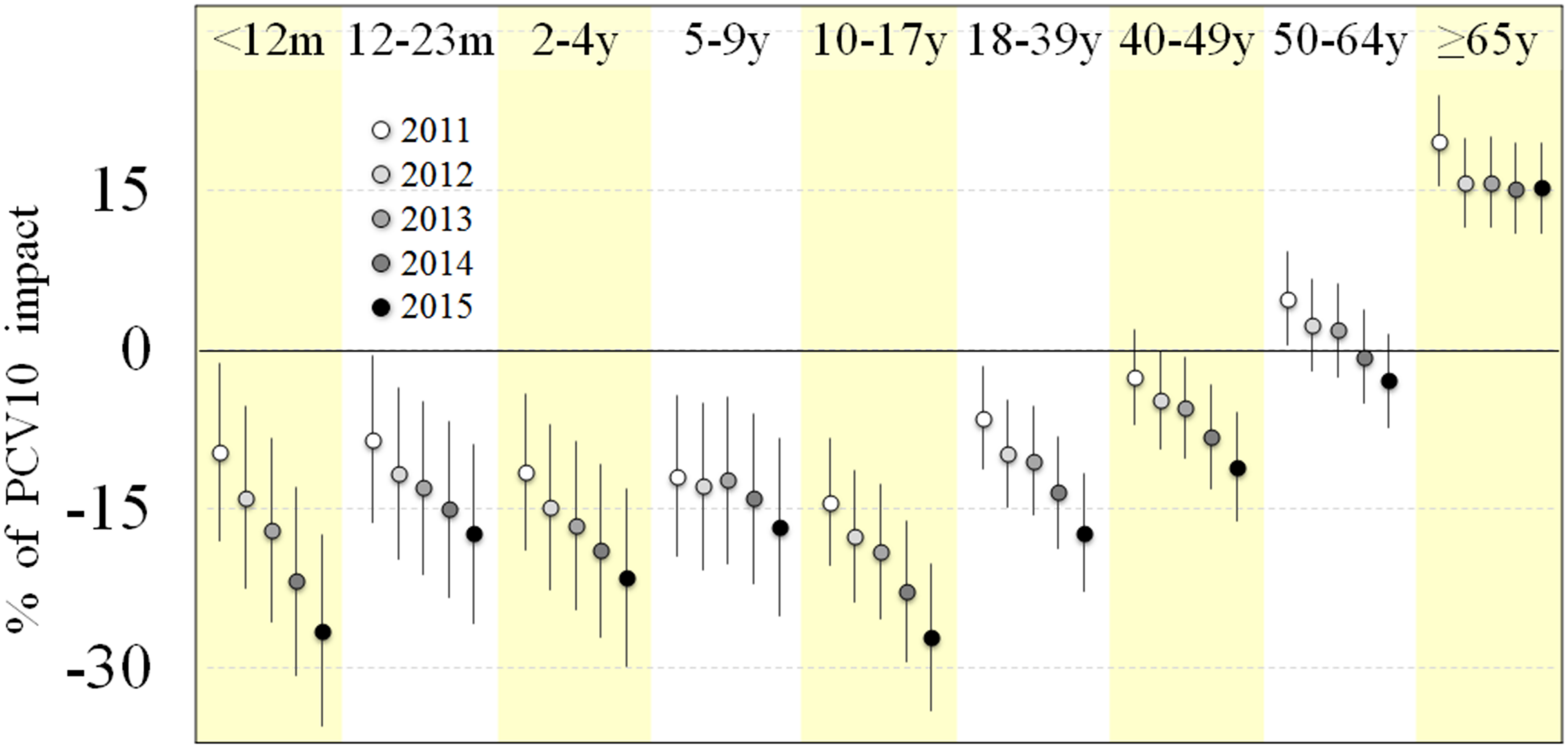
Annual relative percentage change on pneumonia hospitalizations by age-group in the post-vaccination period. Brazil, 2011–2015.

Vaccination impact, as measured by the relative percentage change, increased with the accumulating number of years in the post-vaccination period (Fig 3). For those under 40 years of age, vaccination impact was statistically significant from the very first years of the post-vaccination period. For those aged 40–49, it was statistically significant only from 2013.

### Averted pneumonia hospitalization costs

Considering the predicted number of 3,700,325 pneumonia hospitalizations for the post vaccination period, we estimated that a total of 462,940 pneumonia hospitalizations were averted in Brazil for individuals aged less than 65 years (Table 3). The average cost of pneumonia hospitalization varied by age-group and year, being lower in the 2-4y age-group and higher in adults and the elderly followed by children <12 months of age. Average cost per case in all age-groups was BRL 812 (USD 430.5; $Int 552.6) in 2011, BRL 854 (USD 427; $Int 553.9) in 2012, BRL 880 (USD 378; $Int 533.4) in 2013, BRL 913 (USD 356.3; $Int 528) in 2014, and BRL 936 (USD 243.3; $Int 300.3) in 2015.

**Table 3:** Number of predicted, observed and averted cases, cost per case, and estimated averted costs of hospitalized pneumonia following PCV10 introduction, by age-group. Brazil, 2011–2015.

Year and age-group	Predicted number of cases	Predicted number of cases lower 95% CI	Predicted number of cases upper 95% CI	Observed number of cases	Averted number of cases	Cost per hospitalized pneumonia cases (Reais R$)	Total estimated averted costs of hospitalized pneumonia		
							R$^a^	$Int^b^	USD^c^
2011									
<12 months	116,396	107,676	125,117	108,405	7,991	881	7,042,468	4,790,795	3,732,508
12–23 months	80,641	75,524	85,758	67,987	12,654	724	9,156,434	6,228,867	4,852,910
2–4 years	94,759	88,508	101,010	87,784	6,975	695	4,845,533	3,296,281	2,568,132
5–9 years	51,803	48,478	55,128	44,873	6,930	702	4,867,632	3,311,314	2,579,845
10–17 years	32,383	30,602	34,165	30,089	2,294	758	1,738,852	1,182,893	921,592
18–39 years	79,827	76,268	83,386	72,780	7,047	796	5,610,821	3,816,885	2,973,735
40–49 years	42,058	40,181	43,936	39,939	2,119	880	1,864,932	1,268,661	988,414
50–64 years	73,280	69,865	76,696	78,606	-5,326	949	-67,649,950	-46,020,374	-35,854,473
≥ 65 years	161,803	154,571	169,035	195,594	-33,791	925	-31,256,675	-21,263,044	-16,566,038
Sub-total	732,950	691,672	774,229	726,057	6,893		-63,779,952	-43,387,723	-33,803,375
2012									
<12 months	114,144	105,423	122,864	101,030	13,114	915	12,001,933	7,693,547	6,000,966
12–23 months	78,852	73,735	83,969	63,238	15,614	740	11,551,237	7,404,639	5,775,619
2–4 years	92,411	86,160	98,662	78,628	13,783	716	9,868,628	6,326,044	4,934,314
5–9 years	51,318	47,993	54,643	41,248	10,070	724	7,294,708	4,676,095	3,647,354
10–17 years	32,253	30,471	34,034	27,302	4,951	801	3,965,256	2,541,831	1,982,628
18–39 years	80,748	77,189	84,307	67,402	13,346	837	11,170,602	7,160,642	5,585,301
40–49 years	43,170	41,293	45,047	38,264	4,906	939	4,608,696	2,954,293	2,304,348
50–64 years	75,941	72,525	79,357	76,789	-848	1,008	-854,784	-547,938	-427,392
≥ 65 years	167,139	159,907	174,371	187,117	-19,978	1,005	-20,077,890	-12,870,442	-10,038,945
Sub-total	735,975	694,697	777,254	681,018	54,957		39,528,386	25,338,709	19,764,193
2013									
<12 months	112,477	103,756	121,197	99,481	12,996	929	12,070,685	7,315,567	5,190,394
12–23 months	77,853	72,737	82,970	60,847	17,006	749	12,735,793	7,718,663	5,476,391
2–4 years	91,187	84,936	97,437	75,638	15,549	718	11,167,292	6,768,056	4,801,935
5–9 years	51,074	47,749	54,399	42,238	8,836	732	6,464,418	3,917,829	2,779,700
10–17 years	32,605	30,823	34,386	27,402	5,203	812	4,224,836	2,560,507	1,816,679
18–39 years	82,531	78,972	86,090	68,322	14,209	892	12,671,586	7,679,749	5,448,782
40–49 years	44,430	42,553	46,307	38,523	5,907	966	5,704,981	3,457,564	2,453,142
50–64 years	78,514	75,099	81,930	80,302	-1,788	1,069	-1,911,372	-1,158,407	-821,890
≥ 65 years	172,492	165,260	179,724	201,004	-28,512	1,055	-30,080,160	-18,230,400	-12,934,469
Sub-total	743,162	701,883	784,440	693,757	49,405		33,048,058	20,029,126	14,210,665
2014									
<12 months	110,022	101,302	118,743	90,233	19,789	945	18,700,605	10,809,598	7,293,236
12–23 months	76,167	71,050	81,284	57,030	19,137	766	14,666,597	8,477,802	5,719,973
2–4 years	89,200	82,949	95,451	70,111	19,089	736	14,041,868	8,116,687	5,476,329
5–9 years	50,370	47,045	53,695	37,823	12,547	744	9,338,732	5,398,111	3,642,106
10–17 years	32,367	30,586	34,149	23,624	8,743	863	7,545,209	4,361,392	2,942,632
18–39 years	83,002	79,443	86,561	61,202	21,800	917	19,990,600	11,555,260	7,796,334
40–49 years	45,132	43,255	47,009	34,775	10,357	1,013	10,491,641	6,064,532	4,091,740
50–64 years	80,375	76,960	83,791	75,911	4,464	1,131	5,048,784	2,918,372	1,969,026
≥ 65 years	176,215	168,983	183,447	203,667	-27,452	1,106	-30,361,912	-17,550,238	-11,841,146
Sub-total	742,851	701,572	784,130	654,376	88,475		69,462,124	40,151,517	27,090,228
2015									
<12 months	107,763	99,042	116,483	84,563	23,200	948	21,993,600	11,761,283	5,718,336
12–23 months	74,575	69,458	79,692	53,218	21,357	773	16,508,961	8,828,321	4,292,330
2–4 years	87,309	81,058	93,559	63,390	23,919	743	17,771,817	9,503,645	4,620,672
5–9 years	49,762	46,437	53,088	32,858	16,904	778	13,151,312	7,032,787	3,419,341
10–17 years	32,255	30,474	34,037	19,585	12,670	902	11,428,340	6,111,412	2,971,368
18–39 years	83,839	80,280	87,398	51,707	32,132	983	31,585,756	16,890,779	8,212,297
40–49 years	46,049	44,172	47,926	31,629	14,420	1,065	15,357,300	8,212,460	3,992,898
50–64 years	82,689	79,273	86,105	73,815	8,874	1,139	10,107,486	5,405,073	2,627,946
≥ 65 years	181,146	173,914	188,378	213,812	-32,666	1,089	-35,573,274	-19,023,141	-9,249,051
Sub-total	745,387	704,109	786,666	624,577	120,810		102,331,298	54,722,619	26,606,137

^a^ Brazilian Reais.

^b^ International dollars.

^c^ USD dollar.

When these values were multiplied by the total number of pneumonia hospitalizations averted by age-group, the total averted costs of hospitalized pneumonia cases in children targeted by vaccination (<5 years) was approximately BRL 194.1 million (USD 91.9 million, Int$ 115 million) for the 5 years period after PCV10 introduction (2011–2015). Among age-groups in which PCV10 resulted in decreased pneumonia hospitalizations (0–49 years), the total averted costs of hospitalized pneumonia was BRL 383.2 million (USD 147 million, Int$ 225.2 million) for the period. Roughly half of these averted costs (50.1%) incurred in children targeted by vaccination (<5 years). Interestingly, we observed averted costs hospitalized pneumonia in all age-groups till 49 years of age in all the years analyzed, but only in 2014 and 2015 a significant number of pneumonia cases averted were observed in the 50–64 age-group. In all the study period, the costs of hospitalized pneumonia cases increased in the ≥65 year age-group, as a result of an increase in number of observed cases (Table 3). The overall averted costs for individuals less than 50 years of age considering the 2011–2015 period was BRL 383,199,661 (USD 147,004,281; $Int 225,194,791) (S3 Table).

## Discussion

In this study, we report relevant direct and indirect impact of PCV10 vaccination on pneumonia hospitalization in Brazil. Both impacts were observed since the first year of the post-vaccination period and increased over time. The indirect impact is reported up to the 40–49 years of age-group. Reductions on hospitalizations were also found to translate into significant reductions of the economic burden due to the disease.

We observed a significant burden of pneumonia hospitalizations in all age-groups in Brazil througout the study period, comparable to the rates reported in the US prior to PCV7 introduction [11]. Differently from the US, where the greatest pneumonia burden is represented by the age-group of elderly individuals aged 65 years and over, we found the greatest burden in children aged <12 months, with rates almost four times higher than those observed in children of 2–4 years. This finding underscores the importance of preventive interventions against pneumonia in the first years of life.

The range of the direct impact of PCV10 in hospitalization rates for pneumonia in the cohort of children under two years of age (17.4–26.5%) is similar, albeit slightly lower, to that previously reported in 3 large urban centers in Brazil (23–29%) in the first year after PCV10 introduction [18]. This may be due to a combination of various issues. This previous study had probably better quality of data, as the three urban centers were chosen for this very purpose, and there were also methodological variations, such as the exclusion of duplicate records, among others. Our direct impact estimates are similar to those presented by an active population-based surveillance on pneumonia hospitalization conducted in one city of the Mid-Western region in Brazil, which reports reductions of 12.6% and 14.2% in pneumonia hospitalizations for children aged 2–11 months and 12–23 months, respectively, 3 years after vaccine introduction [37].

In addition to direct effects, our study demonstrates significant indirect effects in the population up to 40–49 years of age group. Bruhn et al. [26], which applied a different time-series methodology to Brazilian hospitalization data up to 2013, also report indirect effects of slightly lower magnitude when compared to ours, up to the age group of 18–39 years of age. In another study in the US, indirect impact was also observed in individuals aged 18–39 years old, but only four years after PCV7 introduction [38]. We believe that the fact that in Brazil the indirect effects were observed from the first years of the post-vaccination period and had an increasing magnitude over the study years may be due to the high PCV10 vaccination coverage attained very quickly throughout the country after vaccination start in Brazil. This contrasts to the relatively low coverage of the first years since PCV7 vaccination started in the US [39].

Differently from what was observed by Griffin et al in US [12], where the indirect effect of PCV7 was found to extend to individuals in the older age groups, we observed increasing trends of pneumonia hospitalizations in elderly individuals aged 65 years and older (15.2%). These increasing rates may represent true increase in disease burden in this age group or may be an artifact due to unmeasured changes in diagnostic and/or coding practices or due to other sources of bias that we were unable to prevent with our methodology.

Similar increases in pneumonia hospitalization rates of elderly individuals were also observed in Scotland as reported by Nair and colleagues six year after PCV7 and PCV13 introduction (2+1 schedule) [40]. In this Scottish study, hospitalizations due to all-cause pneumonia increased from 46%-63% in individuals aged ≥75 years. In Australia, Menzies et al also described no significant changes in hospitalizations in adults above 65 years, six years following the introduction of PCV 7 and PCV13 [41]. In our study, the observed increase in pneumonia hospitalizations in this age-group started long before PCV10 introduction, and vaccine introduction was not able to alter this trend. These findings align with those from previous studies conducted in US which, after 4 years of PCV7 introduction, found no trend reduction in all-cause pneumonia hospitalization rates of individuals 65 years or more [42].

Most studies on the indirect impact of PCV on pneumonia are from countries in which a 3+1 schedule was used. This is also the schedule adopted in Brazil [14]. Evidence of the indirect impact of PCV on pneumonia in countries where 2+1 and 3+0 schedules are used is still lacking. In Uruguay, a study evaluating children under 15 years of age failed to demonstrate the presence of any indirect effect in this age-group, five years after PCV7/PCV13 introduction [43].

The observed economic impact of PCV10 on averted direct costs associated to hospitalized pneumonia is a very important finding. The economic impact following vaccine introduction is perceived by various stakeholders to be of great value in aiding decision-making [44]. Most economic evaluation studies of vaccine introduction are done prior to vaccine introduction. By aggregating an assessment of economic burden to a time-series modeling of vaccine impact, we have estimated the averted costs of all hospitalizations occurring in SUS in Brazil. As innovative analyses of historical observational datasets have been recently recommended as a means to assess the economic impact of vaccination [45], we believe our methods are valuable to assess the post-introduction impact using observed data, and as such provide more robust evidence than models whose assumptions are not driven by data.

Significant costs were averted as a result of PCV introduction in Brazil. In the age groups targeted by the vaccine (0–5 years) a total of BRL 194.1 million (USD 91.9 million, Int$ 115 million) was averted in the post-vaccination period. A similar amount was averted in the age groups of those non-targeted by the vaccine (5–49 years), of BRL 189.1 million (USD 70.5 million, Int$ 110.2 million).

Even without having shown indirect impact in the older age groups, which do represent a significant proportion of the burden of pneumonia in the country, we identified a reduction in costs of hospitalized pneumonia in the 50–64 year age-group, which seems to be more important in 2014. However, for the older age group of individuals 65 years and older, the increase in pneumonia hospitalizations led to an increase in costs of approximatelly BRL 147.4 million (USD 60.6 million; Int$ 88.9 million) in the 5 year period after PCV10 introduction.

There is strong evidence of temporal association between respiratory infections caused by many viral agents and episodes of bacterial pneumonia in the elderly. Studies show that pneumonia is caused by many viral and bacterial infections other than pneumococci serotypes included in PCV10 [46]. It is important to mention that, the population >60 years of age in Brazil has been the target of annual vaccination campaigns against influenza virus for about two decades, with stable vaccination coverage of 85% [16]. This implies that pneumonia hospitalizations in the elderly seem to have increased in recent years in Brazil, despite the high rates of PCV10 vaccination in infants and of the influenza vaccination, which target this population. Little is known about the distribution of viral and bacterial aetiologies causing community acquired pneumonia in the elderly in Brazil. There is also scarce data on which serotypes are responsible for the pneumococcal pneumonias. However, there seems to be an indication that, differently from children, in which the majority of serotypes causing IPD are covered by PCV10, there is a greater diversity in serotypes causing IPD in adults with 50 years and more [47]. As a result, PCV10 vaccination of children may not be affecting at least a proportion of the serotypes causing pneumonia in the elderly. It could be that there is one or more combining reasons for the increase of pneumonia in the elderly, or even just aging of the population.

Our study has some limitations, which should be addressed. Since we used a secondary source of data, which has been originally developed for administrative purposes, the possibility of misdiagnosis of the cause of hospitalization recorded cannot be ruled out. However, a recent study found good agreement between the diagnoses of pneumonia as registered in SIH and those detected by primary data collected though active hospital surveillance [48]. As we used the SIH database without personal identifier, the identification and exclusion of duplicate records could not be performed. Thus, it is possible that the number of hospitalizations may be overestimated as some hospitalization records may represent the same episode of disease in the same patient, registered more than one time. However, we do not believe that this would impact our analyses, as previous studies have demonstrated that the distribution of duplicate records in SIH is randomly distributed in both the pre- and post-vaccination periods [18]. Also, as SIH is the information system for hospitalizations in SUS only, hospitalizations occurring in the supplementary and private healthcare systems were not considered. We do not think that the exclusive use of SIH data reduces the validity of the study, since this database covers all publicly funded hospitalizations in Brazil, and its coverage remained stable throughout the study period [20]. Regarding the averted pneumonia costs, our estimates are conservative as we consider SUS reimbursement costs in the base case, which are significantly lower than costs of pneumonia hospitalization for children in Brazil as reported in the literature [7,49,50]. Studies from the private healthcare sector in Brazil have estimated the costs of pneumonia hospitalization to be 10 times higher than the costs considered in our analysis [51].

## Conclusions

Vaccination with PCV10 five year after its introduction in Brazil was associated with a relevant reduction in all-cause pneumonia hospitalizations and related costs in the target age-groups for vaccination and in unvaccinated individuals aged up to the age-group of 40–49 years, indicating the indirect impact provided by vaccination. Further studies are needed to identify factors associated with the increase in trends of pneumonia hospitalizations in the elderly, considered a matter of concern for public health decision-makers given the rapid aging of the Brazilian population.

## Supporting information

S1 Dataset The dataset generated / analyzed during the current study. (XLSX)

S1 File ICD10 codes for comparison groups according to age-groups. (DOCX)

S2 File R modeling scripts. (DOCX)

S1 Fig Trends in observed (black lines), fit curve (red lines in the pre-vaccination period), and predicted (red lines in the post-vaccination period) hospitalization monthly rates per 100,000 population for the comparison group by age-groups in Brazil. Pre-vaccination period: 2005–2009; Transition period (year of PCV10 introduction): 2010; Post-vaccination period: 2011–2015. Routine infant PCV10 vaccination was introduced through March to September 2010 by the National Immunization Program. The yellow bar represents the transition period which was excluded from the analysis. Gray bar highlights the months excluded of the flu pandemic months (April-October 2009) from the model.
(TIF)

S1 Table Annual number for pneumonia and comparison group hospitalization, and mid-year population estimates by age-group. Brazil, 2005–2015.
(DOCX)

S2 Table Mean percentage error of fitting models of time-series analyses for pneumonia and comparison group hospitalizations rates by age-group. (DOCX)

S3 Table Number of predicted, observed and averted cases, cost per case, and estimated averted costs of hospitalized pneumonia following PCV10 introduction, by age-group. Brazil, 2011–2015.
(DOCX)
