# Correction: Direct and indirect impact of 10-valent pneumococcal conjugate vaccine introduction on pneumonia hospitalizations and economic burden in all age-groups in Brazil: A time-series analysis

**DOI:** 10.1371/journal.pone.0189039

**Published:** 2017-11-30

**Authors:** The PLOS ONE Staff

The abstract of the article is missing. The publisher apologizes for this error. The abstract of the paper is as follows:

***Background*:** Ten-valent pneumococcal conjugate vaccine (PCV10) was introduced in the National Immunization Program of Brazil in March/2010. Although there are recent reports of PCV10 impact on pneumonia hospitalizations, there is still uncertainty regarding the indirect impact in individuals non-targeted by vaccination. We assessed both direct and indirect effect of PCV10 on pneumonia hospitalizations and the impact on the economic burden of pneumonia hospitalizations.

***Methods*:** An interrupted time-series analysis was conducted considering monthly rates of pneumonia hospitalizations and comparison groups, in all age-groups, from January/2005-December/2015. We used records of the National Hospitalizations Information System. Observed pneumonia rates in the post-vaccination period (2011–2015) were compared to predicted rates, should PCV10 had not been introduced. Relative percent difference in rates and its 95% confidence interval were estimated. The number of pneumonia hospitalizations averted by vaccination was calculated as the difference between the predicted and observed cumulative number of pneumonia hospitalizations in the post-vaccination period. The impact of PCV10 on economic burden was presented as averted costs of pneumonia hospitalization.

***Results*:** Significant decrease in rates of pneumonia hospitalization was observed in both children targeted by vaccination (17.4%–26.5%; p<0.01), and in age-groups not targeted by vaccination (11.1%–27.1%, in individuals 10–49 years; p<0.01). In contrast, PCV10 introduction did not alter the increasing trends in pneumonia hospitalization among elderly ≥65 years. A total of 457,564 pneumonia hospitalizations was averted in Brazil for individuals aged <50 years, with a total averted costs of BRL 383.2 million (Int$ 225.2 million, and USD 147 million) for the 5 year period after PCV introduction.

***Conclusion*:** Vaccination with PCV10 5 years after its introduction in Brazil was associated with a relevant reduction in pneumonia hospitalization in the target age-groups, with an indirect effect in individuals aged 10–49 years, and significant reduction in associated economic burden. The increasing trends in pneumonia hospitalization rates in the elderly is a matter of concern for public health and should be further investigated.
