# Supplementary material for: Direct and indirect impact of 10-valent pneumococcal conjugate vaccine introduction on pneumonia hospitalizations and economic burden in all age-groups in Brazil: A time-series analysis
Source: PLoS One. 2017 Sep 7;12(9):e0184204. doi: 10.1371/journal.pone.0184204 (PMC5589174; doi:10.1371/journal.pone.0184204)
Supplement: S1 File — (DOCX) [file pone.0184204.s002.docx]

**S1 File - ICD-10 codes for the comparison groups, according to age-groups.**

For the components of the comparison group, we used all ICD-10 codes, except for the following ones*:

| 1. Chapter F (Mental and behavioural disorders) | other age-groups | children less than 18 years of age | children less than 60 months of age | children less than 24 months of age | children less than 12 months of age |
| --- | --- | --- | --- | --- | --- |
| 1. Chapter J (Diseases of the respiratory system) |  |  |  |  |  |
| 1. Chapter O (Pregnancy, childbirth and the puerperium), |  |  |  |  |  |
| 1. Chapter U (Codes for special purposes) |  |  |  |  |  |
| 1. Chapter I (A00-A09, Intestinal infectious diseases; A18, Tuberculosis of other organs, A40.3, Sepsis due to Streptococcus pneumoniae; B95, Streptococcus and staphylococcus as the cause of diseases classified to other chapters; B97, Viral agents as the cause of diseases classified to other chapters) |  |  |  |  |  |
| 1. Chapter VI (G00-G04, Bacterial meningitis, not elsewhere classified; Meningitis in bacterial diseases classified elsewhere; Meningitis in other infectious and parasitic diseases classified elsewhere; Meningitis due to other and unspecified causes; Encephalitis, myelitis and encephalomyelitis) |  |  |  |  |  |
| 1. Chapter VII (H10, Conjunctivitis; H65, Nonsuppurative otitis media; H66, Suppurative and unspecified otitis media) |  |  |  |  |  |
| 1. Chapter I (B96, Other specified bacterial agents as the cause of diseases classified to other chapters) |  |  |  |  |  |
| 1. Chapter XI (K80, Cholelithiasis) |  |  |  |  |  |
| 1. Chapter XX (V01-Y98, External causes of morbidity and mortality) |  |  |  |  |  |
| 1. Chapter XI (K35, Acute appendicitis) |  |  |  |  |  |
| 1. Chapter XIV (Diseases of the genitourinary system) |  |  |  |  |  |
| 1. Chapter XII (Diseases of the skin and subcutaneous tissue) |  |  |  |  |  |
| 1. Chapter XVI (Certain conditions originating in the perinatal period) |  |  |  |  |  |
| 1. Chapter XVII (Congenital malformations, deformations and chromosomal abnormalities) |  |  |  |  |  |

* Long-stay hospitalizations were also excluded. It is defined in a specific field of the Hospitalization Authorization Form, which includes psychiatric patients, and after Kidney, Liver, Heart, Lung, Bone Marrow and Cornea Transplantation.
