# Supplementary material for: Direct and indirect impact of 10-valent pneumococcal conjugate vaccine introduction on pneumonia hospitalizations and economic burden in all age-groups in Brazil: A time-series analysis
Source: PLoS One. 2017 Sep 7;12(9):e0184204. doi: 10.1371/journal.pone.0184204 (PMC5589174; doi:10.1371/journal.pone.0184204)
Supplement: S2 File — (DOCX) [file pone.0184204.s003.docx]

**S2 File. R modeling scripts.**

# R Project for Statistical Computing: https://www.r-project.org/

# versão R 3.4

### Funções ####

# func tendência, Regressão Linear

calc.tend = function(serie) {

alpha = 0.05 # 5%

n.serie = length(serie)

x.ind = 1 : n.serie # eixo X

lm.res = lm ( serie ~ x.ind ) # Ajuste Linear

lm.coef = lm.res $ coefficients

names(lm.coef) = NULL

tendencia = FALSE

tendencia = summary(lm.res) $ coefficients [2,4] <= alpha

if (is.na(tendencia) | is.null(tendencia)) { tendencia = FALSE }

if (!tendencia) {pos.neg = ''} else {

pos.neg = '(+)'

if (summary(lm.res) $ coefficients [2,1] < 0 ) { pos.neg = '(-)' }

}

pv.lm = round(summary(lm.res) $ coefficients [2,4], digits=4)

pv.lm2 = ifelse(pos.neg=='', '', pv.lm)

summary(lm.res)

}

# func Sazonalidade, Kruskal-Wallis Rank Sum Test

calc.sazon = function(serie) {

periodo = 6 # em meses

alpha2 = .05 # significância

# número de pontos na série

n.serie = length (serie)

# número de grupos

n.grupos = as.integer(n.serie / periodo)

kruskal.g = NULL # inicializa o valor da variável

sazon = FALSE # inicializa o valor da variável

# loop gerando os grupos

for (ind.g in 1:n.grupos) {

kruskal.g = c(kruskal.g, rep(ind.g, periodo))

}

# Kruskal-Wallis Rank Sum Test

temppv = kruskal.test (serie, kruskal.g) $ p.value

# p-valor

sazon.pv = ifelse (!is.na(temppv), round(temppv,6), 0.5)

# significância estatística

sazon = sazon.pv < alpha2 # 90% -> .1

sazon

# p-valor

sazon.pv

}

# func Sazonalidade, Kruskal-Wallis Rank Sum Test

calc.sazon.bimensal = function(serie) {

periodo = 3 # em meses

alpha2 = .05 # significância

# número de pontos na série

n.serie = length (serie)

# número de grupos

n.grupos = as.integer(n.serie / periodo)

kruskal.g = NULL # inicializa o valor da variável

sazon = FALSE # inicializa o valor da variável

# loop gerando os grupos

for (ind.g in 1:n.grupos) {

kruskal.g = c(kruskal.g, rep(ind.g, periodo))

}

# Kruskal-Wallis Rank Sum Test

temppv = kruskal.test (serie, kruskal.g) $ p.value

# p-valor

sazon.pv = ifelse (!is.na(temppv), round(temppv,6), 0.5)

# significância estatística

sazon = sazon.pv < alpha2

sazon

# p-valor

sazon.pv

}

# Função que determina testes de:

# 1) Tendência

# 2) Sazonalidade

# 3) desazonalizar

# bibliotecas

library (stats) # kruskal.test

# Função Tendência, ajuste linear

# novo: resolve se tem tendência

func.tend.teste = function (serie) { # série histórica, Z

alpha = 0.05 # 5%

n.serie.pto = length(serie)

x.ind = 1 : n.serie.pto

lm.res = lm ( serie ~ x.ind )

lm.coef = lm.res $ coefficients

names(lm.coef) = NULL

tendencia = FALSE

tendencia = summary(lm.res) $ coefficients [2,4] <= alpha

if (is.na(tendencia) | is.null(tendencia)) { tendencia = FALSE }

if (!tendencia) {pos.neg = ''} else {

pos.neg = '(+)'

if (summary(lm.res) $ coefficients [2,1] < 0 ) { pos.neg = '(-)' }

}

res = list()

res[[1]] = tendencia

res[[2]] = pos.neg

return (res) # tem tendência?

}

# Função Desazonalizar (tirar tendência)

# novo: leva a pendente da reta para 0

func.tira.tend = function (serie.ag, serie.ant) { # serie.ant

n.serie.pto = length(serie.ag)

x.ind = 1: n.serie.pto

lm.res = lm ( serie.ag ~ x.ind )

lm.coef = lm.res $ coefficients

names(lm.coef) = NULL

ret.a = lm.coef[2] # m = pendente

ret.b = lm.coef[1] # b = cte

y.est = rep (-1, n.serie.pto)

for ( ind.ret in 1 : n.serie.pto ) {

y.est [ind.ret] = serie.ag [ind.ret] - (ret.a * ind.ret + ret.b) + ret.b

}

return (y.est)

} # desazonalizar

# Função Teste de Sazonalidade, Kruskal-Wallis Rank Sum Test

# série histórica, Z

func.sazon.teste = function (serie ) {

periodo = 6 # em meses

alpha2 = .05 # significância

# sazon a partir so 1ro pto?

n.serie = length (serie)

n.grupos = as.integer(n.serie / periodo)

kruskal.g = NULL

sazon = FALSE # ini

for (ind.g in 1:n.grupos) { kruskal.g = c(kruskal.g, rep(ind.g, periodo)) } # gerando os grupos

temppv = kruskal.test (serie, kruskal.g) $ p.value # Kruskal-Wallis Rank Sum Test

sazon.pv = ifelse (!is.na(temppv), temppv, 0.5)

sazon = sazon.pv < alpha2 # 90% -> .1

if ( n.serie >= 36 ) { #

if ( ! sazon ) {

inicio.ag = 3

serie2 = serie [inicio.ag: (n.serie -(periodo-inicio.ag+1) )]

n.serie = length (serie2)

n.grupos = as.integer(n.serie / periodo)

kruskal.g = NULL

sazon = FALSE # ini

for (ind.g in 1:n.grupos) { kruskal.g = c(kruskal.g, rep(ind.g, periodo)) } # gerando os grupos

temppv = kruskal.test (serie2, kruskal.g) $ p.value # Kruskal-Wallis Rank Sum Test

sazon.pv = ifelse (!is.na(temppv), temppv, 0.5)

sazon = sazon.pv < alpha2 # 90% -> .1

}

if ( ! sazon ) {

n.serie = length (serie)

inicio.ag = 5

serie2 = serie [inicio.ag: (n.serie -(periodo-inicio.ag+1) )]

n.serie = length (serie2)

n.grupos = as.integer(n.serie / periodo)

kruskal.g = NULL

sazon = FALSE # ini

for (ind.g in 1:n.grupos) { kruskal.g = c(kruskal.g, rep(ind.g, periodo)) } # gerando os grupos

temppv = kruskal.test (serie2, kruskal.g) $ p.value # Kruskal-Wallis Rank Sum Test

sazon.pv = ifelse (!is.na(temppv), temppv, 0.5)

sazon = sazon.pv < alpha2 # 90% -> .1

}

}

n.serie = length (serie)

return (sazon) # tem sazonalidade?

} # fim da Função Teste de Sazonalidade, Kruskal-Wallis Rank Sum Test

# Função Teste de Sazonalidade, Kruskal-Wallis Rank Sum Test

# série histórica, Z

func.sazon.teste.bimensal = function (serie ) {

periodo = 3 # em meses

alpha2 = .05 # significância

# sazon a partir so 1ro pto?

n.serie = length (serie)

n.grupos = as.integer(n.serie / periodo)

kruskal.g = NULL

sazon = FALSE

for (ind.g in 1:n.grupos) { kruskal.g = c(kruskal.g, rep(ind.g, periodo)) } # gerando os grupos

temppv = kruskal.test (serie, kruskal.g) $ p.value # Kruskal-Wallis Rank Sum Test

sazon.pv = ifelse (!is.na(temppv), temppv, 0.5)

sazon = sazon.pv < alpha2

if ( n.serie >= 18 ) {

if ( ! sazon ) {

inicio.ag = 2

serie2 = serie [inicio.ag: (n.serie -(periodo-inicio.ag+1) )]

n.serie = length (serie2)

n.grupos = as.integer(n.serie / periodo)

kruskal.g = NULL

sazon = FALSE # ini

for (ind.g in 1:n.grupos) { kruskal.g = c(kruskal.g, rep(ind.g, periodo)) } # gerando os grupos

temppv = kruskal.test (serie2, kruskal.g) $ p.value # Kruskal-Wallis Rank Sum Test

sazon.pv = ifelse (!is.na(temppv), temppv, 0.5)

sazon = sazon.pv < alpha2 # 90% -> .1

}

}

n.serie = length (serie)

return (sazon) # tem sazonalidade?

} # fim da Função Teste de Sazonalidade, Kruskal-Wallis Rank Sum Test

# Função Teste do Sinal (Tendência), Cox-Stuart

# divide a série em dois blocos (metade)

# e avalia se há aumento ou diminuição

func.tend.teste.b = function (serie) { # série histórica, Z

alpha = .95 # diferentes dos outros

tendencia = FALSE

n.serie = length (serie) # número de pontos na série histórica

paridade = as.integer(n.serie/2) == n.serie/2 # número de ptos é par?

if (paridade) {tam.c = n.serie/2} else {tam.c = (n.serie+1)/2}

res.dif = serie[1:tam.c] - serie[(tam.c+1):n.serie]

n.mais = sum(res.dif > 0) # número de +

n.menos = sum(res.dif < 0) # número de -

res.t = (abs ( 2 * n.mais - (n.mais + n.menos)) - 1) / sqrt(n.mais + n.menos)

tendencia = res.t > qnorm(alpha, mean = 0, sd = 1, lower.tail = TRUE, log.p = FALSE)

return (tendencia) # tem tendência?

} # fim da Função Teste do Sinal (Tendência), Cox-Stuart

# Função Desazonalizar (tirar tendência)

# 1ra versão: Xi = Xi - X(i-1)

func.tira.tend.b = function (serie.ag, serie.ant) {

res = serie.ag - serie.ant

return (res)

} # desazonalizar

# Procedimento Holt-Winters

#

# O procedimento de ajuste para suavização exponencial de Holt-Winters aditivo

# Uma série temporal Zt pode ser descrita pelos seus componentes de sazonalidade, nível e tendência, assim:

# Z t = µ t + T t + S t + e t onde:

# µ t = componente de nível ou valor média da série, será representado por Zbar;

# T t = componente de tendência da ST;

# S t = componente sazonal da ST; e

# e t = componente de erro aleatório da ST.

#

# As estimativas dos fatores de tendência, nível e sazonal será:

# T(t)= a*( Zbar(t) – Zbar(t-1)) + (1-a)*T^(t-1);

# Zbar(t) = b*(Z(t) – S^(t-r) + (1-b)*(Zbar(t-1) + T^(t-1));

# S(t) = c*(Z(t) – Zbar(t)) + (1-c)*S(t-r); onde:

#

# r = período de sazonalidade ( 12 meses );

# a = parâmetro de tendência;

# b = parâmetro de nível;

# c = parâmetro de sazonalidade.

#

# Os valores iniciais do procedimento são:

# S^(t) = Z(t) / (1/r) * Soma_r( Z(t) ), t = 1,2, ... , r.

# Zbar(r) = (1/r) * Soma_r( Z(t) ), t = 1,2, ... , r.

# T(r)= 0.

#

# As estimativas dos valores futuros da série será:

# Zt(h) = Zbar(t) + h*T^(t) + S^( t+h-s),

# para h = 1,2, ... , r.

#

# O erro será obtido por:

# e(t) = Z^(t) – Z(t), estimado menos observado.

# Cores das linhas:

col.critico = 'red3' # Crítico , vermelho escuro , (205, 000, 000) = (Red, Green, Blue)

col.alerta = 'red1' # Alerta , vermelho claro , (255, 000, 000)

col.atencao = 'yellow2' # Atenção , amarelo escuro , (238, 238, 000)

col.estavel = 'gray' # Estável , sinza , (190, 190, 190)

col.bom = 'skyblue1'# Bom , azul celeste , (135, 206, 255)

col.melhoria = 'green1' # Melhoria , verde claro , (000, 255, 000)

col.excelente = 'green3' # Excelente , verde escuro , (000, 205, 000)

l.graf.cor = c( col.critico, col.alerta, col.atencao, col.estavel, # <--- piora

col.bom, col.melhoria, col.excelente) # ---> melhora

Z = serie_teste

linhas.dp = c(0.995, 1.96, 2.968)

graf.media = mean (Z) # média da série histórica

graf.dp = sd (Z)

l.azul = graf.media + (linhas.dp[1] * graf.dp)

l.verde = graf.media + (linhas.dp[2] * graf.dp)

l.vermelha = graf.media + (linhas.dp[3] * graf.dp)

l.azul.inf = graf.media - (linhas.dp[1] * graf.dp)

l.verde.inf = graf.media - (linhas.dp[2] * graf.dp)

l.vermelha.inf = graf.media - (linhas.dp[3] * graf.dp)

l.graf.val = c(l.vermelha, l.verde, l.azul, graf.media,

l.azul.inf, l.verde.inf, l.vermelha.inf )

n.linhas = length (l.graf.val)

periodo = 6

periodo.sazon = 12

# N=Não(sem), S=Sim(com), alpha=a, beta=b, gama=c

alpha.NN = .25; beta.NN = .5; gama.NN = .25

alpha.SN = .5; beta.SN = .5; gama.SN = .25

alpha.NS = .25; beta.NS = .5; gama.NS = .5

alpha.SS = .5; beta.SS = .25; gama.SS = .5

inclina.lim = 0.75

# Teste de Tendência , inclinação do ajuste linear

res.tendencia.temp = func.tend.teste ( serie = Z )

res.tendencia = res.tendencia.temp [[1]]

res.tendencia.pos.neg = res.tendencia.temp [[2]]

# Se Tendência -> tirar (desazonalizar)

Z.saz = Z

x.ind = (1 : n.ptos.serie) / n.ptos.serie

max.min = max(Z) - min(Z)

max.min = ifelse(max.min != 0, max.min, 1)

serie.ag = (Z - min(Z)) / max.min

lm.res = lm ( serie.ag ~ x.ind )

lm.coef = lm.res $ coefficients

names(lm.coef) = NULL

#if ( abs(lm.coef[2]) > inclina.lim & res.tendencia ) {

if ( res.tendencia ) {

Z.saz = func.tira.tend ( Z , Z49 [ 1:n.ptos.serie ] )

}

# Teste de Sazonalidade, Kruskal-Wallis rank sum test

res.sazon = func.sazon.teste ( serie = Z.saz )

n.0 = sum(Z == 0) # número de pontos=0 na série

#serie.soma = sum(Z) # soma de todos os valores na série (apenas o numerador)

mmsc.hwa.escolha = TRUE

HWA.escolha = 1 # Holt-Winters aditivo

# N=Não(sem), S=Sim(com), alpha=a, beta=b, gama=c

if (!res.tendencia & !res.sazon) {alpha.ag=alpha.NN; beta.ag=beta.NN; gama.ag=gama.NN} # Sem Tend e Sem Saz

if ( res.tendencia & !res.sazon) {alpha.ag=alpha.SN; beta.ag=beta.SN; gama.ag=gama.SN} # Com Tend e Sem Saz

if (!res.tendencia & res.sazon) {alpha.ag=alpha.NS; beta.ag=beta.NS; gama.ag=gama.NS} # Sem Tend e Com Saz

if ( res.tendencia & res.sazon) {alpha.ag=alpha.SS; beta.ag=beta.SS; gama.ag=gama.SS} # Com Tend e Com Saz

# sazon

F.sazon = rep(NA, n.ptos.serie)

F.sazon[1:periodo.sazon] = Z [1: periodo.sazon] / mean(Z [1: periodo.sazon])

# b em t

b.t = rep(NA, n.ptos.serie)

b.t[periodo.sazon] = 0 # ini

# Z médio, Z barra

Z.bar = rep(NA, n.ptos.serie)

Z.bar[periodo.sazon] = mean(Z [1: periodo.sazon])

# Z estimado, Z chapeo

Z.hat = rep(NA, n.ptos.serie ) # + periodo)

# estimação do pasado

for (i.t in (periodo.sazon+1):n.ptos.serie) {

Z.bar[i.t] = (beta.ag * (Z[i.t]

- F.sazon [(i.t - periodo.sazon)])

+ (1-beta.ag) * (Z.bar[(i.t - 1)] + b.t[(i.t - 1)]))

F.sazon[i.t] = gama.ag * (Z[i.t] - Z.bar[i.t]) + (1-gama.ag) * F.sazon[(i.t - periodo.sazon)]

b.t[i.t] = alpha.ag * (Z.bar[i.t] - Z.bar[(i.t - 1)]) + (1-alpha.ag) * b.t[(i.t - 1)]

Z.hat[i.t] = Z.bar[i.t] + F.sazon[i.t] + b.t[i.t]

}

# erro

erro = Z.hat [1:n.ptos.serie] - Z # vetor

# MPE, Mean Percentage Error (MPE, Erro Percentual Médio)

erro_mpe[ind_ij] = abs( sum(erro / Z * 100, na.rm = TRUE) / n.ptos.serie )

# modelo ajuste

# serie_teste0 - dad reais, s/ correção pandemia

# serie_teste - dad reais, c/ correção pandemia

# Z = serie_teste

# Z.hat = modelo aj = ajuste

modelo_aj = data.frame (serie_teste0, serie_teste, Z, Z.hat ) # Z.hat [1:n.ptos.serie]

n.mes.junto = 1 # mensal

# ano ini e fim

ano.ini = 2005

ano.fim = 2015

ano.fim.aj = 2009 # ajuste, antes da interrupção

ano.ini.prev = 2011 # previsão, após a interrupção

serie_nome.geral = 'Taxas mensais para internação por 100.000'

# período

periodoST = paste(ano.ini,'-',ano.fim, sep='' )

nPtosAno = 12 / n.mes.junto

periodo = nPtosAno / n.mes.junto

periodo.sazon = nPtosAno

meses2.ind = 1:nPtosAno * n.mes.junto - (n.mes.junto-1)

meses = c('Jan','Fev','Mar','Abr','Mai','Jun','Jul','Ago','Set','Out','Nov','Dez')

meses2 = meses[meses2.ind]

mes.meio.ano = meses2 [periodo]

ano.mes.ini = paste(ano.ini, '01', sep='')

ano.mes.fim = paste(ano.fim, '12', sep='')

ano.mes.fim.aj = paste(ano.fim.aj, '12', sep='')

n.anos.aj = ano.fim.aj - ano.ini +1

n.anos.interrup = ano.ini.prev - ano.fim.aj -1

n.anos.prev = ano.fim - ano.ini.prev +1

n.prev = nPtosAno * n.anos.prev # em meses

### faixa etária (TODAS) =====

# nomes no arquivo .doc (NOME)

fxs.et.doc.nome = c(

'menor 12 meses'

,'12 a 23 meses'

,'2 a 4 anos'

,'5 a 9 anos'

,'10 a 17 anos'

,'18 a 39 anos'

,'40 a 49 anos'

,'50 a 64 anos'

,'65 anos e mais'

)

### faixa etária (TODAS) =====

# nomes no arquivo .csv (código)

fxs.et.csv.cod = c(

'<12m'

,'12-23m'

,'2-4anos'

,'5-9anos'

,'10-17anos'

,'18-39anos'

,'40-49anos'

,'50-64anos'

,'>=65anos'

)

# compara nome vs código:

data.frame( fxs.et.doc.nome, fxs.et.csv.cod)

fx.et.ordem = fxs.et.csv.cod

n.fx.et.ordem = length (fx.et.ordem)

# a mesma para todas as faixas etárias

ser.teste.ordem = rep( 'TX_PN', length(fxs.et.csv.cod))

# Grupos de comparação:

ser.ctrl.ordem =

rep('tx_comparison' ,length(fxs.et.csv.cod))

comparacoes.fxEt.Test.Ctrl = data.frame ( fx.et.ordem, ser.teste.ordem, ser.ctrl.ordem )

comparacoes.fxEt.Test.Ctrl

# códigos e nomes para tds as ser test e ctrl

serie_test_ctrl.cods = c(

'tx_pn'

,'tx_comparison'

)

serie_test_ctrl.nomes = c(

'pneumonia'

,'comparison'

)

data.frame(serie_test_ctrl.cods, serie_test_ctrl.nomes)

# tenho nomes de tds os cód ser test

length(ser.teste.ordem)

sum(is.element(ser.teste.ordem, serie_test_ctrl.cods ))

sum(!is.element(ser.teste.ordem, serie_test_ctrl.cods ))

reg.nome = 'Brasil'

# todas as faixas

fxIdade.nome = fxs.et.csv.cod

fxIdade.nome2 = fxs.et.doc.nome

meses = c('Jan','Fev','Mar','Abr','Mai','Jun','Jul','Ago','Set','Out','Nov','Dez')

setwd (pasta_dados)

dir()

# Carrega o arquivo texto CSV com os dados

dad = read.table (file = arq,

sep=';',

header=TRUE,

stringsAsFactors=FALSE,

dec='.')

str(dad)

regs = 'Brasil'

regs.n = length( regs )

# faixa etária

fxs = unique(sort(dad $ fx_etaria))

fxs.n = length( fxs)

sum(!is.element( fxs, fxIdade.nome))

sum(!is.element( fxIdade.nome, fxs))

# faixas etárias em análise

fxs = fx.et.ordem # fxIdade.nome

fxs.n = length( fxs)

fx1 = 0

fx2 = ''

test_pv = 0

test_pv_sn = ''

test_m = 0

test_sazon_pv =0

test_sazon =''

real1114 = 0

prev1114 = 0

area_pv = 0

area_med = 0

ic = 0

explica = 0

alpha2 = .05 # significância 5%

ind_ij = 0

real1114_2011 = 0

prev1114_2011 = 0

area_pv11 = 0

area_med11 = 0

pdf(file = arq.pdf , paper='a4r', width=11, height=8 )

idade.ag = fx.et.ordem[ind.fx] # código

fx.et.csv.ind = which (is.element(fxs.et.csv.cod, idade.ag))

idade.nome.ag = fxs.et.doc.nome [fx.et.csv.ind]

# ser test

serie_teste.cod = ser.teste.ordem[ind.fx]

ser.cod.ind = which (is.element(serie_test_ctrl.cods, serie_teste.cod))

serie_teste.nome0 = serie_test_ctrl.nomes [ser.cod.ind]

serie_teste.nome = paste(

'Taxas mensais de hospitalização por ', serie_teste.nome0 ,' por 100 mil', sep='')

# ser ctrl

serie_controle.cod = ser.ctrl.ordem[ind.fx]

ser.cod.ind = which (is.element(serie_test_ctrl.cods, serie_controle.cod))

serie_controle.nome0 = serie_test_ctrl.nomes [ser.cod.ind]

serie_controle.nome = paste(

'Taxas mensais de hospitalização por ', serie_controle.nome0 ,' por 100 mil', sep='' )

ind_ij = ind_ij +1

ind.reg.ag = which ( dad $ AnoMes >= ano.mes.ini & dad $ AnoMes <= ano.mes.fim

& dad $ fx_etaria == fxs[ind.fx]

)

dad2 = dad [ind.reg.ag, ]

# ordem por data AAAAMM

ind.anomes = order ( dad2 $ AnoMes )

dad3 = dad2[ind.anomes,]

# série completa

indSerAll = which ( dad3 $ AnoMes >= ano.mes.ini & dad3 $ AnoMes <= ano.mes.fim )

dad3 = dad3[indSerAll,]

# série pré interrupção

indSerPre = which ( dad3 $ AnoMes >= ano.mes.ini & dad3 $ AnoMes <= ano.mes.fim.aj )

dad4 = dad3[indSerPre,]

x.legenda = paste( meses[ as.numeric(substring(dad3 $ AnoMes,5) )], substr(dad3 $ AnoMes,3,4 ), sep=' ')

ind.jan = which (substr(x.legenda,1,3) =='Jan')

x.legenda.ano = substring(dad3 $ AnoMes,1,4)

ind.jun = which (substr(x.legenda,1,3) =='Jun')

# interrupção

ind.int = which (is.element(x.legenda,c('Jan 10','Dez 10')))

# serie (teste)

ind.col = which(is.element(dimnames(dad)[[2]], serie_teste.cod ))

serie_teste = dad4 [, ind.col]

serie_teste_all = dad3 [, ind.col]

serie_teste0 = serie_teste

serie_teste_all0 = serie_teste_all

n.ptos.serie = length(serie_teste)

# estimação do futuro

n.ptos.futuro = length(serie_teste_all) - length(serie_teste)

prev = serie_teste

# desconsiderando pandemia2 (Abr a Out / 2009) , versão 2017-05-05

# abril-outubro de 2009 : "Abr 09" "Mai 09" "Jun 09" "Jul 09" "Ago 09" "Set 09" "Out 09"

pand.mes = c("Abr 09", "Mai 09", "Jun 09", "Jul 09", "Ago 09", "Set 09", "Out 09" )

pand.ind = which (is.element(

x.legenda [ 1: length(prev) ],

pand.mes))

x.legenda [pand.ind]

pand.n = length (pand.ind)

serie_teste = prev

prev.ind = (1: n.ptos.futuro) + n.ptos.serie

serie.teste2 = c( serie_teste_all, prev.mm [prev.ind] )

ylimite = c(min(serie.teste2) * .9, max(serie.teste2) * 1.1) # pelas faixas IC

ylimite0 = c(0, ylimite[2])

# Tendência ###

tend.teste = calc.tend ( serie_teste ) # dad reais

tend.teste.coef = tend.teste $ coefficients

# serie

serie_teste_all.n = length( serie_teste_all )

ser_test_fut = serie_teste_all [ serie_teste_all.n -c(n.prev:1) + 1 ]

tend.teste.fut = calc.tend ( ser_test_fut )

tend.teste.coef.fut = tend.teste.fut $ coefficients

######## sozanalidade ####

sazon.teste = calc.sazon ( serie_teste ) # dad reais, c/ correção pandemia

#sazon.controle = calc.sazon ( serie_controle )

fx1[ind_ij] = idade.ag

fx2[ind_ij] = idade.nome.ag

test_pv[ind_ij] = round(tend.teste.coef [2,4], 4) # Pr(>|t|)

test_pv_sn[ind_ij] = ifelse (test_pv[ind_ij] < alpha2, 'Sim', 'Não')

test_m[ind_ij] = round(tend.teste.coef [2,1], 4) # Estimate

test_sazon_pv[ind_ij] = sazon.teste

test_sazon[ind_ij] = ifelse (sazon.teste < alpha2, 'Sim', 'Não')

print (' ')

print ('===========================')

print( paste( ind_ij, fx1[ind_ij], fx2[ind_ij] , sep=' - '))

## Modelagem ##

identifica = paste ( '_Br_', test.ctrl, '_05-a-15_FxEt_', ind.fx, '.csv', sep='')

# salva o arquivo texto CSV com serie teste real

write.table (fut.real,

file = paste('./resultados/Real', identifica, sep=''),

sep=';', row.names = FALSE, dec='.')

# salva o arquivo texto CSV com serie teste prev

write.table (fut.prev,

file = paste('./resultados/Prev_futuro', identifica, sep=''),

sep=';', row.names = FALSE, dec='.')

# salva o arquivo texto CSV com serie teste real, hist comp

write.table (serie_teste_all,

file = paste('./resultados/Real_histComp', identifica, sep=''),

sep=';', row.names = FALSE, dec='.')

# prev pasado

write.table (Z.hat,

file = paste('./resultados/Prev_pasado', identifica, sep=''),

sep=';', row.names = FALSE, dec='.')

# ajuste modelo

write.table (modelo_aj,

file = paste('./resultados/Modelo_aj', identifica, sep=''),

sep=';', row.names = FALSE, dec='.')

# resultados prev (pós-vacina) :: 2011 a 2015

real1114 [ind_ij] = round(sum(fut.real),2)

prev1114 [ind_ij] = round(sum(fut.prev),2)

real.prev.dif = fut.real / fut.prev -1

############################# graf ajuste, graf aj ajuste, Ymin=0

### 2011 a 2015 ##########################################

result = data.frame(

, fx1

, fx2

, test_pv

, test_pv_sn

, test_m

, test_sazon_pv

, test_sazon

, real1115

, prev1115

, area_pv

, area_med

, ic

, explica

)

# salva o arquivo texto CSV com os dados

write.table (result,

file = paste( ident2, '______.csv', sep=''),

sep=';',

row.names = FALSE,

col.names = c(

,'Faixa_et'

,'Faixa_et'

,'Tend_pv'

,'Tend'

,'Tend_m'

,'Sazon_pv'

,'Sazon'

, 'real_2011a2015'

, 'prev_2011a2015'

, 'area_pvalor'

, 'area_media'

, 'IC'

, 'explica'

),

dec='.')

### 2011 ##########################################

# 2011

result2011 = data.frame(

, fx1

, fx2

, real1115_2011

, prev1115_2011

, area_pv11

, area_med11

)

# salva o arquivo texto CSV com os dados - 2011

write.table (result2011,

file = paste( ident2, '2011.csv', sep=''),

sep=';',

row.names = FALSE,

col.names = c(

,'Faixa_et'

,'Faixa_et'

, 'real_2011'

, 'prev_2011'

, 'area_pvalor'

, 'area_media'

),

dec='.')
