# Supplementary figures and images for: Direct and indirect impact of 10-valent pneumococcal conjugate vaccine introduction on pneumonia hospitalizations and economic burden in all age-groups in Brazil: A time-series analysis
Source: PLoS One. 2017 Sep 7;12(9):e0184204. doi: 10.1371/journal.pone.0184204 (PMC5589174; doi:10.1371/journal.pone.0184204)

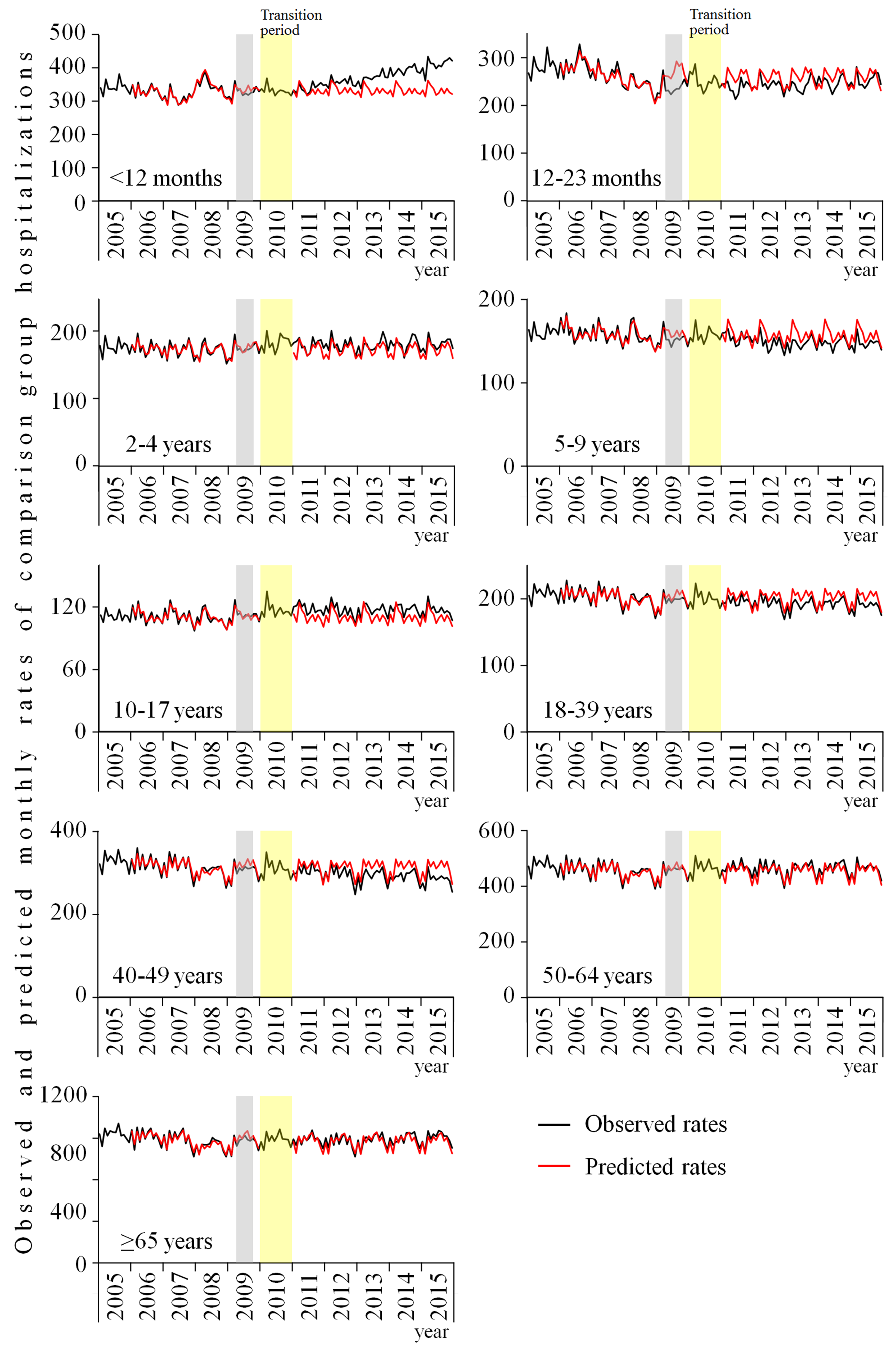

Supplement: S1 Fig — Pre-vaccination period: 2005–2009; Transition period (year of PCV10 introduction): 2010; Post-vaccination period: 2011–2015. Routine infant PCV10 vaccination was introduced through March to September 2010 by the National Immunization Program. The yellow bar represents the transition period which was excluded from the analysis. Gray bar highlights the months excluded of the flu pandemic months (April-October 2009) from the model. (TIF) [file pone.0184204.s004.tif]
