# Supplementary material for: Direct and indirect impact of 10-valent pneumococcal conjugate vaccine introduction on pneumonia hospitalizations and economic burden in all age-groups in Brazil: A time-series analysis
Source: PLoS One. 2017 Sep 7;12(9):e0184204. doi: 10.1371/journal.pone.0184204 (PMC5589174; doi:10.1371/journal.pone.0184204)
Supplement: S1 Table — Brazil, 2005–2015. (DOCX) [file pone.0184204.s005.docx]

**S1 Table. Annual number for pneumonia and comparison group hospitalizations, and mid-year population estimates by age-group. Brazil, 2005-2015.**

| **Age-group** | **2005** | **2006** | **2007** | **2008** | **2009** | **2010** | **2011** | **2012** | **2013** | **2014** | **2015** |
| --- | --- | --- | --- | --- | --- | --- | --- | --- | --- | --- | --- |
| ***Pneumonia*** | | | | | | | | | | | |
| <12 months | 128,655 | 130,970 | 125,511 | 119,987 | 131,131 | 113,252 | 108,405 | 101,030 | 99,481 | 90,233 | 84,563 |
| 12-23 months | 89,698 | 91,303 | 88,223 | 82,048 | 86,791 | 79,818 | 67,987 | 63,238 | 60,847 | 57,030 | 53,218 |
| 2-4 years | 102,970 | 105,579 | 104,958 | 96,382 | 104,286 | 97,511 | 87,784 | 78,628 | 75,638 | 70,111 | 63,390 |
| 5-9 years | 54,677 | 57,811 | 53,673 | 51,881 | 58,921 | 52,193 | 44,873 | 41,248 | 42,238 | 37,823 | 32,858 |
| 10-17 years | 33,223 | 35,276 | 33,004 | 30,395 | 40,432 | 33,691 | 30,089 | 27,302 | 27,402 | 23,624 | 19,585 |
| 18-39 years | 81,229 | 79,904 | 76,176 | 72,944 | 94,134 | 80,311 | 72,780 | 67,402 | 68,322 | 61,202 | 51,707 |
| 40-49 years | 38,790 | 39,345 | 37,541 | 37,957 | 45,846 | 42,675 | 39,939 | 38,264 | 38,523 | 34,775 | 31,629 |
| 50-64 years | 60,580 | 62,470 | 62,530 | 65,541 | 77,735 | 78,516 | 78,606 | 76,789 | 80,302 | 75,911 | 73,815 |
| ≥ 65 years | 127,608 | 134,701 | 140,257 | 145,463 | 170,880 | 182,716 | 195,594 | 187,117 | 201,004 | 203,667 | 213,812 |
| Total | 717,430 | 737,359 | 721,873 | 702,598 | 810,156 | 760,683 | 726,057 | 681,018 | 693,757 | 654,376 | 624,577 |
| ***Comparison group*** | | | | | | | | | | | |
| <12 months | 109,580 | 104,204 | 96,733 | 105,723 | 98,038 | 97,459 | 97,980 | 100,965 | 102,736 | 106,995 | 109,504 |
| 12-23 months | 98,630 | 98,789 | 90,934 | 80,813 | 78,144 | 81,238 | 75,666 | 75,598 | 73,945 | 74,238 | 73,938 |
| 2-4 years | 195,661 | 191,003 | 185,372 | 179,971 | 179,298 | 185,123 | 179,174 | 174,331 | 170,742 | 169,818 | 165,682 |
| 5-9 years | 271,485 | 271,383 | 263,041 | 255,208 | 247,430 | 252,698 | 246,105 | 232,544 | 229,315 | 228,165 | 223,947 |
| 10-17 years | 367,580 | 368,667 | 367,995 | 358,788 | 368,604 | 383,645 | 388,633 | 380,949 | 382,475 | 385,299 | 378,370 |
| 18-39 years | 1,649,789 | 1,669,413 | 1,668,200 | 1,621,017 | 1,632,230 | 1,686,543 | 1,675,108 | 1,670,635 | 1,660,247 | 1,706,921 | 1,688,236 |
| 40-49 years | 864,794 | 872,346 | 881,447 | 860,754 | 883,470 | 921,992 | 924,950 | 920,725 | 927,410 | 947,419 | 937,098 |
| 50-64 years | 1,184,490 | 1,209,100 | 1,239,465 | 1,236,685 | 1,297,536 | 1,382,236 | 1,429,487 | 1,450,720 | 1,501,442 | 1,562,805 | 1,586,399 |
| ≥ 65 years | 1,344,830 | 1,372,651 | 1,385,970 | 1,360,849 | 1,438,725 | 1,521,051 | 1,561,145 | 1,581,769 | 1,644,053 | 1,710,141 | 1,765,164 |
| Total | 6,086,839 | 6,157,556 | 6,179,157 | 6,059,808 | 6,223,475 | 6,511,985 | 6,578,248 | 6,588,236 | 6,692,365 | 6,891,801 | 6,928,338 |
| ***Mid-year population estimates*** | | | | | | | | | | | |
| <12 months | 2967479.2 | 2917052.4 | 2866625.6 | 2816198.8 | 2765771.9 | 2715345.1 | 2664918.3 | 2614491.5 | 2564064.7 | 2513637.8 | 2463211.0 |
| 12-23 months | 2947280.4 | 2897223.3 | 2847166.1 | 2797109.0 | 2747051.9 | 2696994.7 | 2646937.6 | 2596880.4 | 2546823.3 | 2496766.1 | 2446709.0 |
| 2-4 years | 9192860.4 | 9033219.7 | 8873578.9 | 8713938.2 | 8554297.4 | 8394656.7 | 8235016.00 | 8075375.2 | 7915734.5 | 7756093.7 | 7596453.0 |
| 5-9 years | 15769069.1 | 15610452.1 | 15451835.1 | 15293218.1 | 15134601.1 | 14975984.0 | 14817367.0 | 14658750.0 | 14500133.0 | 14341516.0 | 14182899.0 |
| 10-17 years | 27792020.1 | 27738985.0 | 27685950.0 | 27632914.9 | 27579879.9 | 27526844.8 | 27473809.7 | 27420774.7 | 27367739.6 | 27314704.6 | 27261669.5 |
| 18-39 years | 66499292.3 | 67315768.9 | 68132245.4 | 68948722.0 | 69765198.6 | 70581675.1 | 71398151.7 | 72214628.3 | 73031104.9 | 73847581.4 | 74664058.0 |
| 40-49 years | 22008631.1 | 22570763.7 | 23132896.2 | 23695028.7 | 24257161.3 | 24819293.8 | 25381426.4 | 25943558.9 | 26505691.4 | 27067824.0 | 27629956.5 |
| 50-64 years | 20951300.7 | 21739619.9 | 22527939.0 | 23316258.1 | 24104577.2 | 24892896.4 | 25681215.5 | 26469534.6 | 27257853.8 | 28046172.9 | 28834492.0 |
| ≥ 65 years | 11973445.0 | 12391567.1 | 12809689.1 | 13227811.2 | 13645933.2 | 14064055.3 | 14482177.3 | 14900299.4 | 15318421.4 | 15736543.5 | 16154665.5 |
