# Supplementary material for: Direct and indirect impact of 10-valent pneumococcal conjugate vaccine introduction on pneumonia hospitalizations and economic burden in all age-groups in Brazil: A time-series analysis
Source: PLoS One. 2017 Sep 7;12(9):e0184204. doi: 10.1371/journal.pone.0184204 (PMC5589174; doi:10.1371/journal.pone.0184204)
Supplement: S2 Table — (DOCX) [file pone.0184204.s006.docx]

**S2 Table. Mean percentage error of fitting models.**

| Age-group | Pneumonia | Comparison group |
| --- | --- | --- |
| <12 months | 0.16 | 0.05 |
| 12-23 months | 0.07 | 0.10 |
| 2-4 years | 0.03 | 0.06 |
| 5-9 years | 0.06 | 0.06 |
| 10-17 years | 0.12 | 0.06 |
| 18-39 years | 0.22 | 0.05 |
| 40-49 years | 0.12 | 0.05 |
| 50-64 years | 0.01 | 0.04 |
| ≥ 65 years | 0.11 | 0.06 |
