# Supplementary material for: Direct and indirect impact of 10-valent pneumococcal conjugate vaccine introduction on pneumonia hospitalizations and economic burden in all age-groups in Brazil: A time-series analysis
Source: PLoS One. 2017 Sep 7;12(9):e0184204. doi: 10.1371/journal.pone.0184204 (PMC5589174; doi:10.1371/journal.pone.0184204)
Supplement: S3 Table — Brazil, 2011–2015. (DOCX) [file pone.0184204.s007.docx]

**S3 Table. Overall number of predicted, observed and averted cases, cost per case, and estimated averted costs of hospitalized pneumonia following PCV10 introduction, by age-group. Brazil, 2011-2015.**

| Age-group | Predicted number of cases | Observed number of cases | Averted number of cases | Total estimated averted costs of hospitalized pneumonia | | |
| --- | --- | --- | --- | --- | --- | --- |
|  |  |  |  | R$^a^ | $Int^b^ | USD^c^ |
| <12 months | 560,802 | 483,712 | 77,090 | 71,809,291 | 42,370,790 | 27,935,440 |
| 12-23 months | 388,088 | 302,320 | 85,768 | 64,619,022 | 38,658,292 | 26,117,223 |
| 2-4 years | 454,866 | 375,551 | 79,315 | 57,695,138 | 34,010,713 | 22,401,382 |
| 5-9 years | 254,327 | 199,040 | 55,287 | 41,116,802 | 24,336,136 | 16,068,346 |
| 10-17 years | 161,863 | 128,002 | 33,861 | 28,902,493 | 16,758,035 | 10,634,899 |
| 18-39 years | 409,947 | 321,413 | 88,534 | 81,029,365 | 47,103,315 | 30,016,449 |
| 40-49 years | 220,839 | 183,130 | 37,709 | 38,027,550 | 21,957,510 | 13,830,542 |
| 50-64 years | 390,799 | 385,423 | 5,376 | -55,259,836 | -39,403,274 | -32,506,783 |
| ≥ 65 years | 858,795 | 1,001,194 | -142,399 | -147,349,911 | -88,937,265 | -60,629,649 |
| Total | 3,700,325 | 3,379,785 |  | 180,589,915 | 96,854,249 | 53,867,850 |

^a^  Brazilian Reais

^b^ International dollars

^c^ USD dollar
